# Disparities in Area Socioeconomic Development and Pediatric Cancer Survival in Romania—A National Pediatric Registry Study on Multiple Geographic Levels

**DOI:** 10.3390/cancers18162627

**Published:** 2026-08-14

**Authors:** Jenna Zabroski, Mihaela Bucurenci, Megan A. Healey, Anca Colita, Amr S. Soliman

**Affiliations:** 1 Cancer Epidemiology Education in Special Populations (CEESP), City University of New York, New York, NY 10031, USA; 2 Department of Epidemiology, Boston University School of Public Health, Boston, MA 02118, USA; 3 Romanian National Pediatric Oncology and Hematology Registry, Romanian Society of Pediatric Oncology and Hematology, 022328 Bucharest, Romania; 4 Department of Pediatrics, Faculty of Medicine, “Carol Davila” University of Medicine and Pharmacy, 020021 Bucharest, Romania; 5 Department of Community Health and Social Medicine, City University of New York School of Medicine, New York, NY 10031, USA

**Keywords:** pediatric cancer, cancer survival, socioeconomic disparities, regions, counties, community marginalization, urban, rural, Romania

## Abstract

Background/Objectives: Socioeconomic indicators and geographic factors influence pediatric cancer outcomes, but evidence in Romania is limited. Methods: This retrospective cohort study included 6247 patients aged 0–19 years diagnosed with cancer and recorded in the Romanian National Pediatric Oncology and Hematology Registry between 1 January 2010, and 31 December 2024. Kaplan–Meier analysis was used to estimate the survival probabilities across four strata of regional and county socioeconomic categorization and two strata of community marginalization status. Unadjusted and multivariable Cox proportional hazards models estimated hazard ratios (HRs) and 95% confidence intervals (CIs), adjusting for sex, age group, primary cancer type (ICCC-3), tumor behavior, and geographical residence. Subgroup analyses assessed the association between rurality and pediatric cancer survival, irrespective of community marginalization status. Results: Survival probabilities were consistently lower among patients residing in more socioeconomically disadvantaged regions, counties, and marginalized communities (log-rank *p* ≤ 0.0001). In the most deprived strata, 5-year survival for regions, counties, and communities was 69.86% (95% CI: 67.49–72.10), 67.47% (95% CI: 63.32–71.26), and 65.51% (95% CI: 61.33–69.35), respectively. In adjusted models, residence in the least deprived regions (HR = 0.783, 95% CI = 0.744–0.952) and counties (HR = 0.749, 95% CI = 0.620–0.905) was associated with improved survival, a 22% and 25% lower risk of death, respectively, compared with residence in the most deprived categories. Community marginalization was associated with lower survival outcomes in unadjusted analyses, but was not significant after adjustment. Rural residence was associated with a 44% higher risk of death (HR = 1.436, 95% CI = 1.304–1.582), with a 5-year survival of 66.76% (95% CI: 64.91–68.53) among rural patients, compared with 75.93% (95% CI: 74.28–77.48) in urban patients. Conclusions: This is Romania’s first pediatric cancer survival study to evaluate persistent social and geographical disparities. Survival outcomes were consistently lower in more socioeconomically disadvantaged regions and counties, while the findings suggest that rural residence may explain the observed differences in survival at the community level. Policymakers and health systems in Romania should focus on covering pediatric cancer patients with appropriate proximity services across the entire national territory, thus enabling all patients to get timely access to quality care.

## Simple Summary

Pediatric cancer survival in Romania may be influenced by patient socioeconomic indicators and places of residence. Little is known about how childhood cancer survival in Romania varies across regions, counties, and communities with different levels of socioeconomic deprivation. Using national cancer registry data on 6247 children and adolescents diagnosed with cancer in 2010–2024, this study aimed to investigate survival outcomes by socioeconomic indicators. Survival probabilities and hazard ratios (HRs) were calculated to compare survival across several strata at the regional, county, and community levels. Results showed that children from more socially disadvantaged regions and counties had worse survival outcomes, with 5-year survival of 69.86% in disadvantaged regions and 67.47% in disadvantaged counties (HR = 0.783 and 0.749, respectively; *p* < 0.0001). Children living in rural areas experienced significantly poorer survival outcomes than children living in urban areas, with a 5-year survival of 66.76% compared to 75.93% (HR = 1.436, *p* < 0.0001), while rurality itself appears to be associated with survival, irrespective of marginalization status. Overall, these findings highlight geographic and socioeconomic disparities in pediatric cancer survival differences in Romania.

## 1. Introduction

Pediatric cancer is a leading cause of mortality among children and adolescents globally. Over the past several decades, pediatric cancer survival has increased in many European countries; however, variations in survival outcomes persist between countries and regions. Across Europe, childhood cancer survival exceeds 80% in high-income countries, yet disparities remain between Eastern and Western European regions [1,2,3,4]. These differences have been documented in the EUROCARE-6 study, both in adults and children and are a major concern addressed by current European Union policies [5].

In Romania, an Eastern European country with a total population of approximately 18.8 million [6], national population-based cancer registration is still in development. Pediatric cancer represents only a small proportion of all cancer diagnoses (<1%), with approximately 400 newly recorded diagnosed cases in 2025 [7]. Despite how rare pediatric cancer is, it exerts a high societal impact and has a high profile on the public agenda. In 2010, the Romanian National Pediatric Oncology and Hematology Society (RNPOHS) established a national pediatric cancer registry in Romania, which marked an improvement in national surveillance of childhood cancer incidence, treatment, and survival outcomes. A 2024 pediatric cancer survival study in Romania showed that for the 2020–2017 diagnosed cohort, survival was lower than the European average, with an estimated 5-year survival of 73% compared with about 81% across Europe for children aged 0–14 years. In addition, rural patients experience poorer survival outcomes than urban patients, with reported 5-year survival of 69% in rural areas and 78% in urban areas. Romania also continues to face territorial disparities in socioeconomic development, access to specialized healthcare, and transportation access. These findings suggest that there may be survival differences across broader regional, county, and community socioeconomic contexts [8,9].

Several population-based registry studies have investigated whether socioeconomic disadvantage and geographic residence are associated with pediatric cancer survival. In the United States, retrospective cohort analyses using SEER and state cancer registry data have included children and adolescents aged 0–19 years diagnosed over multi-year periods (typically spanning one to two decades) and have tested the hypothesis that neighborhood-level deprivation is associated with inferior survival outcomes [10]. These studies characterized socioeconomic context using territorial, area-level measures such as the Area Deprivation Index (ADI) or other census tract– and block group–derived indicators [11,12,13]. Other U.S.-based registry studies have evaluated whether rural residence or distance to pediatric oncology centers influences survival, using county- or tract-level geographic classifications within similar population-based cohorts [14,15,16].

A Children’s Oncology Group cohort evaluated household poverty during maintenance therapy for acute lymphoblastic leukemia using federal poverty thresholds, focusing on relapse risk within a clinical trial population [17]. Other U.S. registry-based analyses have used census-tract–level deprivation indices, which differ from national territorial approaches by emphasizing household- or small-area-level exposures and, in some cases, specific cancer subtypes rather than population-wide pediatric cohorts [18,19,20,21,22].

In Europe, nationwide registry-based cohort studies from Denmark, Germany, Greece, and the United Kingdom have examined children and adolescents diagnosed with cancer over extended calendar periods, often spanning multiple decades. These studies have tested whether individual-level parental socioeconomic factors (e.g., education, occupation, income) or area-level deprivation measures are associated with survival differences [23,24,25,26,27,28]. Multi-country European analyses have further assessed geographic and socioeconomic disparities at national or regional levels using population-based cancer registries. Overall, prior research has primarily evaluated socioeconomic disadvantage at a single geographic level—either individual or territorial—with limited investigations simultaneously examining disparities across multiple administrative levels within a single national pediatric cancer registry framework [29,30,31].

Nationwide population-based registry studies have more commonly assessed area-level deprivation using administrative units such as municipalities or regions. A recent German study linked municipality-level socioeconomic deprivation to childhood cancer survival over a 20-year period [24]. In Hungary, long-term registry analyses (e.g., 1971–2015) have evaluated survival and incidence trends in childhood leukemia across regions, often within the context of major socioeconomic transitions [32]. These studies are characterized by extended historical follow-up and territorial comparisons between regions, contrasting with U.S. trial-based or household-level approaches and highlighting broader structural inequalities rather than individual poverty measures [15,19,24,32,33].

Despite the growing body of literature, there is substantial heterogeneity when it comes to defining and measuring socio-economic disadvantage and area-level deprivation across studies. Existing research evaluates socioeconomic context using composite indicators or proxy measured constructed from locally available indicators [10,13,34,35,36]. At the national level in Romania, there is a lack of consistent data at different geographic units of analysis. As a result, there is no single standardized measure that can be applied across all administrative levels in Romania [9,37,38]. Therefore, this study aimed to validate and further explore previously observed geographic disparities in pediatric cancer survival in Romania. The study also examined whether differences in area-level socioeconomic development were associated with pediatric cancer survival across multiple administrative levels: regional, county, and community.

## 2. Materials and Methods

### 2.1. Data Sources and Data Collection

This retrospective cohort study was conducted in Romania. Pediatric cancer information was obtained from the Romanian National Pediatric Oncology and Hematology Registry (RNPOHR) founded by the Romanian National Society of Pediatric Oncology and Hematology. RNPOHR is a database that collects data on childhood cancers in Romania from all pediatric oncology facilities operating across the national territory. RNPOHR data span to 2010, with cancer cases reported by trained personnel using forms based on the European Network of Cancer Registers standard dataset. The collected information includes sex, age at diagnosis, place of residence, survival status, date of diagnosis, tumor behavior, primary cancer classification, treatment modality, and follow-up status [39]. Tumors are classified according to the International Classification of Diseases and Oncology, 3rd edition (IDC-O-3), and the International Classification of Childhood Cancer, 3rd edition (ICCC-3). There are 12 primary ICCC-3 cancer types [40,41].

For each case, the retrieved information is aggregated by personal identification number. Each case of cancer is verified, coded, and finalized by a trained cancer registry reviewer. Each report in the registry also undergoes automatic validation using Version 2.2.8 of the JRC-ENCR Quality Check Software. RNPOHR estimated coverage is about 80% of the total number of new pediatric cancer cases in Romania. Patients who are 15+ years can be treated in adult facilities, whilst there are also situations of new cases, seeking cross-border care without utilizing the national pediatric cancer care network. Vital status is obtained by the Registry, upon request from the Office of Vital Statistics, for cases in the Registry database. 93.5% of cases are microscopically verified [8,39].

Territorial units for area socioeconomic development analysis were based on the European NUTS classification—Nomenclature of Territorial Units for Statistics, a geocode standard for referencing the administrative divisions of European member countries for statistical purposes. EUROSTAT (the statistical office of the European Union) established a hierarchy of three NUTS levels numbered from 1 to 3. Below these are the Local Administrative Units (LAUs) [42]. For Romania, the levels are: NUTS-1 with 4 macroregions; NUTS-2 with 8 regions; NUTS-3 with 41 counties plus the capital city Bucharest and 3181 local administrative units (LAUs) (communes, municipalities and cities)- hereafter designated as “community level” [43,44].

RNPOHR collects information on patients’ place of residence at NUTS-2 and NUTS-3 and LAU level classified according to the Informatic Systemic of the Register of Territorial-Administrative Units (SIRUTA), which codes towns, villages, and administrative units in Romania and distinguishes between urban and rural territories. SIRUTA coding is maintained by the National Institute of Statistics in Romania [45].

Publicly available socioeconomic indicators were derived from national sources to characterize deprivation levels at the regional and county levels, primarily from the National Institute of Statistics Database and from numerous national studies and reports published during the period covered by our study [6,9,44,46,47,48,49,50,51,52,53]. At the regional and county levels, data were collected from the Population and Housing Census (2021) and the annual statistical reports conducted by the National Institute of Statistics [54,55]. Data on the number and distribution of pediatric beds came from the National Institute of Public Health (2017) [56]. Data on the 0–17 age population and the working age population (15–64 years) was also collected from the annual statistical reports through the National Institute of Statistics [55]. Community-level marginalization status was characterized from a national report developed under the EU Operational Programme for Administrative Capacity [38]. Multiple dimensions of socio-economic disadvantage were used to determine whether a given community was marginalized.

Data were fully anonymized, while community-level information was retrieved by authorized registry personnel in compliance with personal data protection regulations applicable to rare diseases.

### 2.2. Study Population

The study population consisted of individuals aged 0–19 years who were diagnosed with cancer between 1 January 2010, and 31 December 2024. Patients were excluded if they were not Romanian residents or if they had missing survival geographic data. Patients with non-malignant tumors except for those of the central nervous system, as well as those with multiple cancers, were also excluded. After applying the inclusion and exclusion criteria, 6247 individuals were included in the descriptive analysis, and 6150 were included in the survival analyses.

To estimate 1-, 3-, and 5-year survival, patients were followed from the date of diagnosis until censoring or death. The last day of the follow-up period is 31 December 2024, or the date of death, whichever comes first. Patients without matched death records for the entire follow-up period were considered alive.

### 2.3. Exposure Variables

Overall, prior research has focused on either individual-level SES or single-level territorial measures. In contrast, our study applies a multidimensional deprivation construct across multiple administrative levels within the same national registry, enabling direct evaluation of geographic granularity in pediatric cancer survival [57]. Socioeconomic disadvantage was defined at three geographic levels: regional, county, and community, using an approach that ensures consistency across levels given the available data in Romania. Standardized socioeconomic deprivation indices are not uniform across geographic levels in Romania, so we selected comparable indicators at each level as proxies, consistent with the dimensions most frequently included in area deprivation characterizations, such as poverty, education, access to health facilities, infrastructure development, and living conditions. This approach examines associations between area-level socioeconomic deprivation at regional, county, and community levels, and the pediatric cancer survival outcomes.

At the regional and county levels, data were collected using publicly available data to reflect the multidimensional nature of socioeconomic deprivation in Romania. At the community level, socioeconomic data for individual indicators were not available across localities in Romania. Therefore, community marginalization status and criteria were assessed using a report from the Operational Programme Administrative Capacity, a program funded by the European Union with the goal of improving public administration efficiency [38].

The indicators selected for this study are intended to capture the multidimensional nature of socioeconomic deprivation and area-level development in Romania. Indicators includes economic conditions, education level, infrastructure measures, and access to specialized pediatric care, which are commonly used in socioeconomic deprivation index frameworks [34,38]. A standardized approach to measure a socioeconomic deprivation index is not consistently available across geographic levels in Romania. Therefore, we conceptually compared across levels based on publicly available data to provide a comprehensive representation of socioeconomic conditions in relation to pediatric cancer outcomes. These measures reflect area-level deprivation effects, not individual household socioeconomic disadvantage. Area deprivation captures the structural and systemic characteristics of place, including regional development, health system access, transport infrastructure, service availability, and access to care. All of these may influence pediatric cancer outcomes [38]. Using indicators across multiple domains provides a thorough characterization of socioeconomic context and allows us to examine associations between area-level deprivation and pediatric cancer survival outcomes.

#### 2.3.1. Regional Socioeconomic Deprivation Categorization

Six socioeconomic indicators were used at the regional level: the proportion of the population at risk of poverty or social exclusion (AROPE), number of pediatric hospital beds per 1000 pediatric population (0–17 years), gross domestic product (GDP) per inhabitant (USD), minimum income for inclusion rate per 1000 working age population (15–64 years), proportion of the population with low educational attainment, and the percentage of homes connected to a sewer [54,55,56,58,59,60,61]. Pediatric hospital bed availability was standardized using the population aged 0–17 years based on the available national data.

A social deprivation index is inherently multifactorial and incorporates multiple measures including economic resources, access to care and services, infrastructure, and poverty. AROPE was included as a standardized European indicator of socioeconomic deprivation. AROPE is the rate of being at risk of poverty or social exclusion defined by meeting at least one of the following criteria: (1) income below 60% of the median income, (2) severely materially and socially deprived, and (3) living in a household with very low work intensity [58]. The number of pediatric hospital beds was chosen as an indicator to represent the access to specialized pediatric healthcare, a critical component of healthcare accessibility [56]. The GDP per inhabitant was included to capture territorial differences in economic development and the distribution of wealth across Romania. It is a good indicator to assess territorial disparities for the overall economic development of a given area of Romania. The minimum income for inclusion rate is a proxy for poverty risk and economic vulnerability as it captures several dimensions of the poverty risk or poverty exposure of the population. It covers both those who are unemployed and those whose income is insufficient to meet the basic living commodities in daily life. Rates were calculated based using the working age population (17–65 years) because it captures families at the age of being more likely to raise children and adolescents, aligning with the pediatric focus of the study [6]. Educational attainment was represented by the proportion of the population of the low educational attainment. This reflects both the working market disadvantage and health literacy understanding, including the capacity to take charge of a child’s health situation or condition [6]. Lastly, the percentage of homes connected to the sewage system includes components of both infrastructure development and housing conditions. Covering the sanitation of a home, the place in which a child lives, and the level of infrastructural development of the community itself allows for a greater assessment of area-level deprivation. Taken together, these indicators represent socioeconomic deprivation that is relevant to pediatric cancer survival outcomes [54,55,56,58,59,60,61,62].

**Figure 1:**
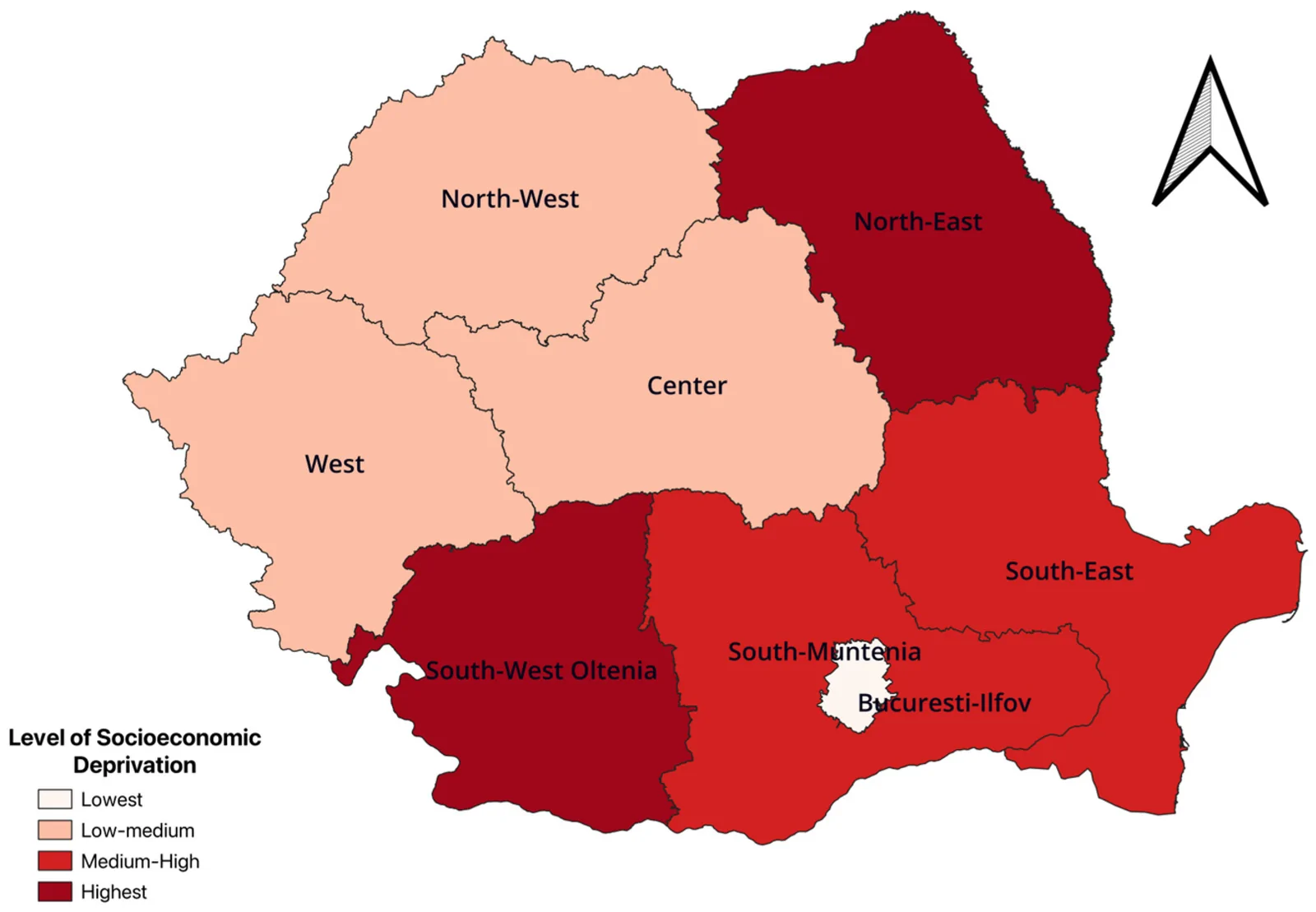
Map of Regional Socioeconomic Deprivation Categorization in Romania.

Histograms and choropleth maps with graduated color symbology were generated using QGIS to examine the distribution of each indicator and to identify appropriate class break points for further categorization. The six indicators were combined to characterize regional socioeconomic deprivation. Regions were grouped into four categories (lowest, low-medium, medium-high, and highest) based on class breaks derived from the distribution of combined values. “Lowest” represented the least socially deprived regions, and “highest” represented the most socially deprived regions. A natural breaks (Jenks) classification method was used to determine the cutoff points [63]. Using this approach identifies natural groupings within the data to minimize within-group variance and to show the variation in socioeconomic conditions across regions. Maps were visually assessed to ensure that the categorization aligned with known regional development patterns in Romania. Figure 1 shows how the eight economic development regions were categorized by socioeconomic deprivation.

#### 2.3.2. County Socioeconomic Deprivation Categorization

Five socioeconomic indicators were used at the county level: the number of pediatric hospital beds per 1000 pediatric population (0–17 years), GDP per inhabitant (measured in USD), minimum income for inclusion rate per 1000 working age population (15–64 years), low educational attainment rate, and the percentage of homes connected to a sewer. AROPE was not used for the county level because public data at this level was unavailable. Pediatric hospital bed availability was standardized using the population aged 0–17 years based on the available national data. Maintaining consistent indicators across levels allowed for consistent characterization of socioeconomic deprivation and allows for comparisons to be made across geographic levels [47,54,55,56,59,60,61,62].

**Figure 2:**
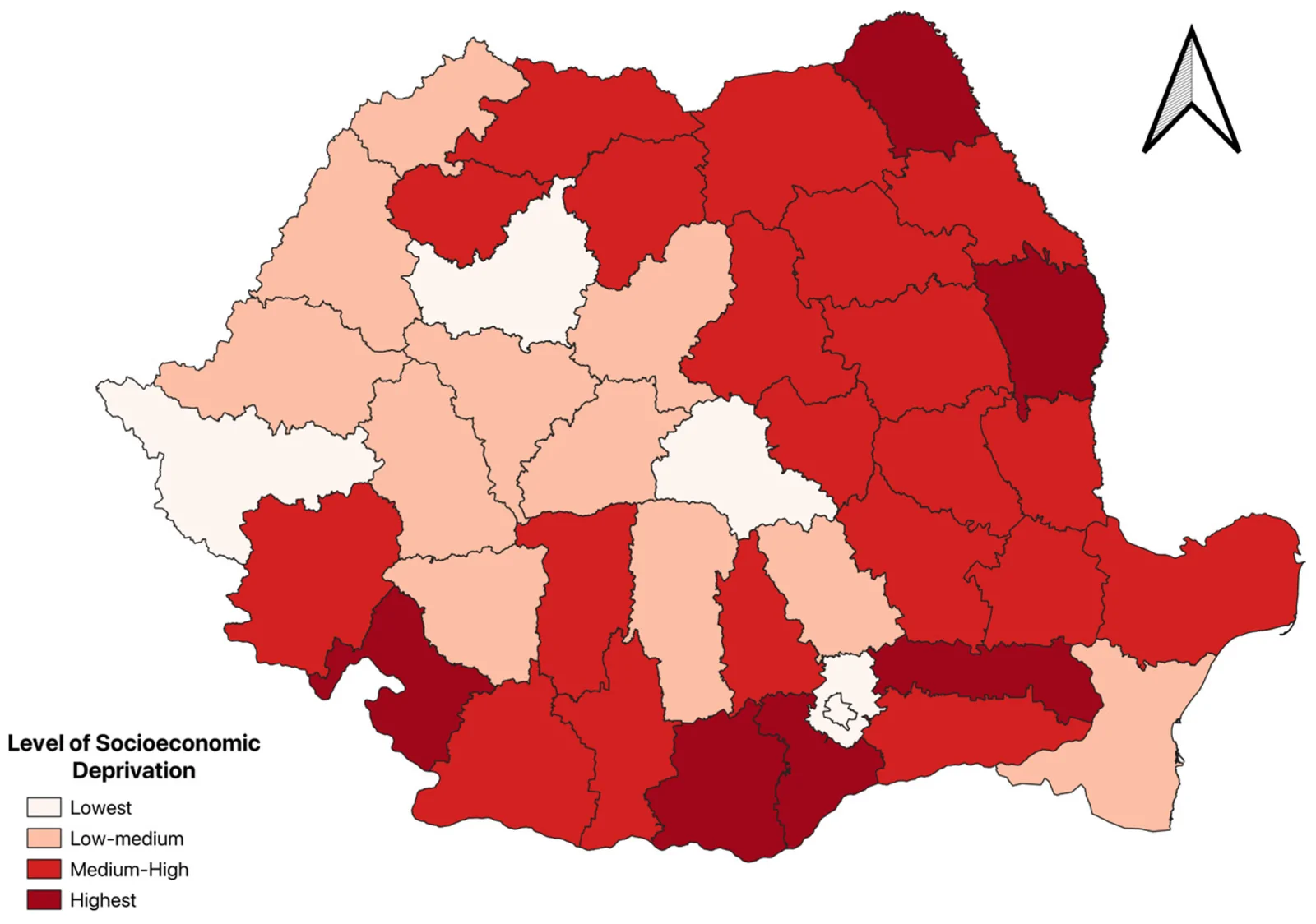
Map of County Socioeconomic Deprivation Categorization in Romania.

The same framework being applied across geographic levels allows for comparability between regional and county measures. Figure 2 illustrates the classification of Romanian counties according to socioeconomic deprivation.

#### 2.3.3. Community Marginalization Status Categorization

At the community level, socioeconomic indicator data were not publicly available in Romania; therefore, marginalization status was used instead. To conduct this analysis, a report from the Operational Programme for Administrative Capacity, a program funded by the European Union with the goal of improving public administration efficiency, was used. This report provides a standardized national framework for identifying marginalized communities based on multiple dimensions of socioeconomic disadvantage [38]. Unlike the regional- and county-level deprivation measures, the community marginalization index was based on this existing national framework.

Marginalization is a multidimensional socioeconomic phenomenon that implies cumulative disadvantages across multiple dimensions within a territorially delimited area. This is based on a nationally standardized framework developed under the Operational Programme for Administrative Capacity. The framework evaluates community marginalization using eight dimensions of socioeconomic disadvantage [38]. These dimensions were aggregated into a composite marginalization index to further classify socioeconomic disadvantage. Communities were defined as administrative localities (communes, towns, municipalities) and sublocal zones within localities (urban neighborhoods, rural villages). This is the smallest geographic unit for which marginalization data was available [38]. Communities were classified as marginalized if their composite marginalized index value fell within the highest quintile (upper 20%) of deprivation according to the framework used. Assessing marginalization in this manner is multifactorial, as health marginalization is rarely isolated and co-exists with other factors, including poverty, poor housing, lack of access to transportation, and low levels of education. The framework aligns the European Union and national strategies on social inclusion [64,65].

Based on data collected from city halls through questionnaires, the national framework groups relevant indicators of marginalization were grouped into eight dimensions: access to public utilities, exposure to pollution sources, infrastructure and access to services, transport, poverty, aging demographics and housing, access to hospital and medical services, access to education, and ethnic segregation [6]. These dimensions are combined into the composite marginalization index used in the present study [6,50,52,56,60].

Pediatric cancer cases recorded by the registry were linked to community marginalization status using SIRUTA coding [45]. After linkage, 598 cases recorded by the registry were classified as residing in marginalized communities.

### 2.4. Outcome Definition

The primary outcome is overall pediatric cancer survival estimated at 1-, 3-, and 5-years. Survival was measured in months from the date of diagnosis to the end of follow-up (31 December 2024) or the date of a reported death, whichever came first. Survival probabilities were estimated at 1-, 3-, and 5-year intervals at the regional, county, and community analysis levels. Survival was also estimated for the two community-level subgroup analyses.

### 2.5. Covariates

The study included the following demographic variables collected in the RNPOHR registry: age (years) and sex (male/female), as well as geographical place of residence (urban/rural)with territorial distribution at 3 NUTS levels: economic development regions (North-East, Bucharest-Ilfov, Center, North-West, South-East, South-Mutenia, South-West Oltenia, and West), county of residence (one of 42 counties, including the municipality of Bucharest), and city/commune/village level of residence (one of 3181 territorial administrative units). Patients’ clinical information recorded in the Registry had been retrieved from patient medical records, including the 12 primary ICCC-3 cancer types [40], tumor behavior (borderline and in situ vs. malignant, and reported treatment (unimodal: no treatment, surgery, radiotherapy, and systemic therapy; multimodal: combination of the reported treatments).

### 2.6. Statistical Analysis

Descriptive statistics were used to summarize the study population overall (0–19 years) and stratified by age group (0–14 years and 15+ years). Means and standard deviations were reported for continuous variables. Frequencies and percentages were used to summarize categorical variables. Analysis was performed across multiple geographical levels: regional, county, and community. At the regional and county levels, baseline characteristics were compared across four categories (lowest, low-medium, medium-high, and highest) of socioeconomic indicator categories. At the community level, marginalization status was divided into two categories: marginalized and non-marginalized, with the cut-off set at the upper quintile of the marginalization continuum, as per the data source methodology. Chi-square tests or Fisher’s exact tests as appropriate, were used to evaluate patients’ characteristics by age group, sex, geographical place of residence, tumor behavior, and primary ICCC-3 cancer type [40]. Differences in mean age were evaluated using one-way ANOVA. Data collected was analyzed using SAS Version 9.4, and statistical significance was assessed at a two-tailed significance level of 0.05.

#### Survival Analysis

In the examined literature, survival analyses have primarily relied on Kaplan–Meier estimation and multivariable Cox proportional hazards models applied within registry-based or clinical trial cohorts [11,12,13,17]. Trial-based analyses occasionally incorporated time-varying covariates to capture changes in individual poverty status, whereas registry studies generally adjusted for demographic and clinical confounders and, when applicable, clustering at a single geographic level.

Similarly, European nationwide registry studies have predominantly used Cox regression models, sometimes complemented by relative survival approaches in population-based settings [24,32]. Eastern European analyses often emphasized long-term survival trends and regional comparisons using standard time-to-event techniques [3,8,66,67,68,69,70,71]. However, across both U.S. and European research, statistical modeling has typically been conducted at a single socioeconomic or territorial level, without simultaneous multi-level survival analysis within a coherent framework [4,5,10,72].

In our study, survival probabilities were analyzed at 1-, 3-, and 5-years using the Kaplan–Meier method for each level of analysis (regional, county, and community). Survival time was defined as the time from diagnosis to death, measured in months. Patients were censored at the end of the follow-up period (31 December 2024) or at last known contact. Log-rank tests assessed differences between survival curves. Kaplan–Meier curves are shown in the results for the regional, county, and community analyses.

Unadjusted and multivariate-adjusted Cox proportional hazards regression models were used to estimate hazard ratios (HRs) and 95% confidence intervals (CIs) for socioeconomic indicators categorized at the regional, county, and community levels. Separate models were fit for each geographic level of analysis, using the most advantaged category as the reference group. Multivariable analyses adjusted for sex, age group, tumor behavior, geographic place of residence, and primary cancer type (ICCC-3). Tumor behavior and primary cancer type were included because they are well-established clinical prognostic factors associated with pediatric cancer survival. Age was modeled categorically using predefined pediatric age groups because these categories are clinically meaningful and consistent with pediatric oncology reports. Covariates were selected based on prior literature and their established clinical relevance.

Two subgroup analyses were conducted to explore potential confounding by rurality at the community level. In the community marginalization analysis, the majority of marginalized cases were deemed rural, so the first subgroup analysis compared survival between marginalized and non-marginalized communities among rural patients only. The second subgroup analysis compared survival between rural and urban communities, irrespective of marginalization status. Marginalization status was not adjusted for to assess the differences in survival outcomes between urban and rural areas. Kaplan–Meier techniques and unadjusted/multivariate-adjusted Cox proportional regression models were used to estimate survival in these subgroup analyses.

Age-stratified sensitivity analyses were also performed separately among patients aged 0–14 years and 15–19 years and are presented in the Supplementary Materials (Tables S1 and S2 and Figures S1–S3).

## 3. Results

### 3.1. Study Population Characteristics

The current study includes 6247 patients aged 0–19 years diagnosed with cancer and reported to the cancer registry diagnosed between 1 January 2010, and 31 December 2024 (Table 1). In the overall cohort, mean age at diagnosis is 8.20 ± 5.81 years with about 56.01% being male. Overall, 27.98% of patients died during the study period. Approximately half of the cohort resided in urban areas (51.43% urban vs. 48.39% rural), with about 10% of patients in the entire cohort living in a marginalized community. With respect to socioeconomic deprivation categories, 28.25% of patients came from the most deprived region category and 9.96% of patients came from the most deprived county category. Most tumors were malignant (96.93%) with the most common cancer types being leukemias, myeloproliferative diseases and myelodysplastic diseases (30.14%); lymphomas and reticuloendothelial neoplasms (15.45%); and CNS and miscellaneous intracranial and intraspinal neoplasms (14.58%).

Among patients receiving one treatment type, 33.74% received surgery, 10.61% received radiotherapy, and 78.28% received systemic therapy (chemotherapy and/or immunotherapy). Among patients receiving multimodal therapy, systemic therapy was the most frequent component (35.17%), followed by surgery in combination (32.22%).

### 3.2. Baseline Characteristics by Socioeconomic and Geographic Categories

#### 3.2.1. Characteristics by Regional Socioeconomic Category

Baseline characteristics differed across socioeconomic categorization of regions based on deprivation level (Table 2). In this analysis, deprivation levels increase across socioeconomic categories, with the lowest category representing the least deprived regions and the highest category the most deprived areas. Of the cohort, 696 patients were in the lowest category and 1765 in the highest. Mean age varied significantly across categories (*p* < 0.0001) with the distribution of age groups varying significantly by region (*p* = 0.0002). As deprivation level increases, so does the proportion of rural residences with the highest category including 57.73% of rural residences compared to the lowest category with 11.64% (*p* < 0.0001). Tumor behavior (*p* = 0.0210) and primary cancer type (*p* < 0.0001) both differed significantly across categories.

#### 3.2.2. Characteristics by County Socioeconomic Category

Among pediatric cancer patients, characteristics varied by county-level socioeconomic deprivation, ranging from least (lowest) to most deprived (highest) (Table 3). The majority of patients lived in counties with medium-high deprivation (41.88%), followed by low-medium (27.37%), lowest (20.79%), and highest deprivation (9.96%). Mean age varied significantly (*p* < 0.0001), and the distribution of age groups also varied significantly by county deprivation (*p* = 0.0002). Rural residence increased significantly with higher levels of deprivation, with 24.56% in low counties versus 61.25% in high counties (*p* < 0.0001). Tumor behavior (*p* < 0.0001) and primary cancer type (*p* = 0.0212) also varied significantly by county-level indicators of socioeconomic deprivation.

#### 3.2.3. Characteristics by Community Marginalization Status

In the community marginalization analysis, 598 patients (9.57%) resided in a most-marginalized community (Table 4). On average, patients in marginalized communities were significantly older with a mean age of 9.74 ± 5.91 years vs. 8.03 ± 5.77 years for non-marginalized communities (*p* < 0.0001). There were also significant differences by age group (*p* < 0.0001), but no significant difference by sex. Marginalization status is strongly associated with rurality, with 97.66% of marginalized patients residing in rural communities compared with 42.26% of non-marginalized patients residing in rural communities (*p* < 0.0001). There were significant differences by tumor behavior (*p* < 0.0001), but the distributions of primary cancer types did not differ significantly.

### 3.3. Kaplan–Meier Survival Analysis

**Figure 3:**
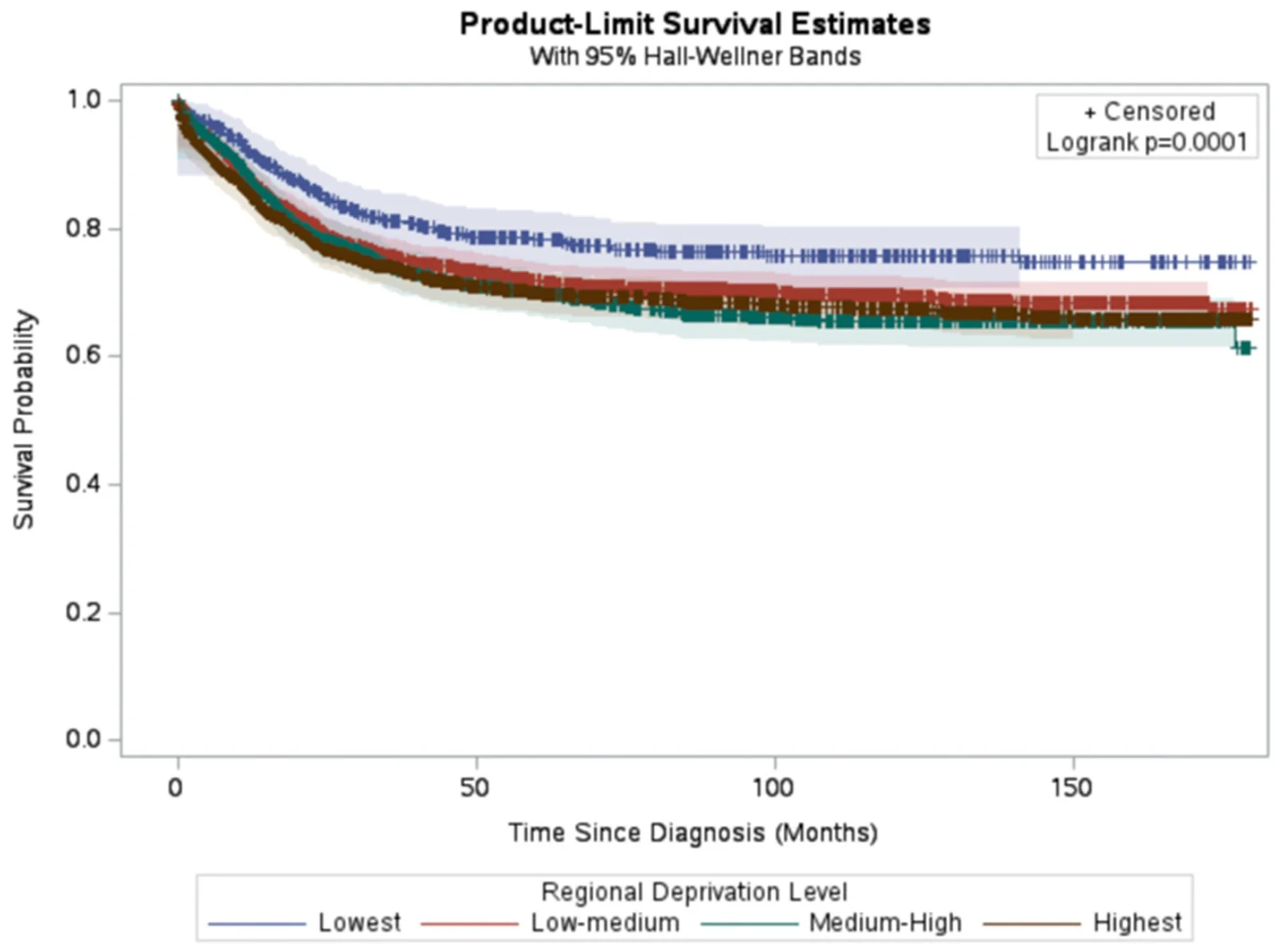
Survival Function by Socioeconomic Categorization of Regions Based on Deprivation Level.

**Figure 4:**
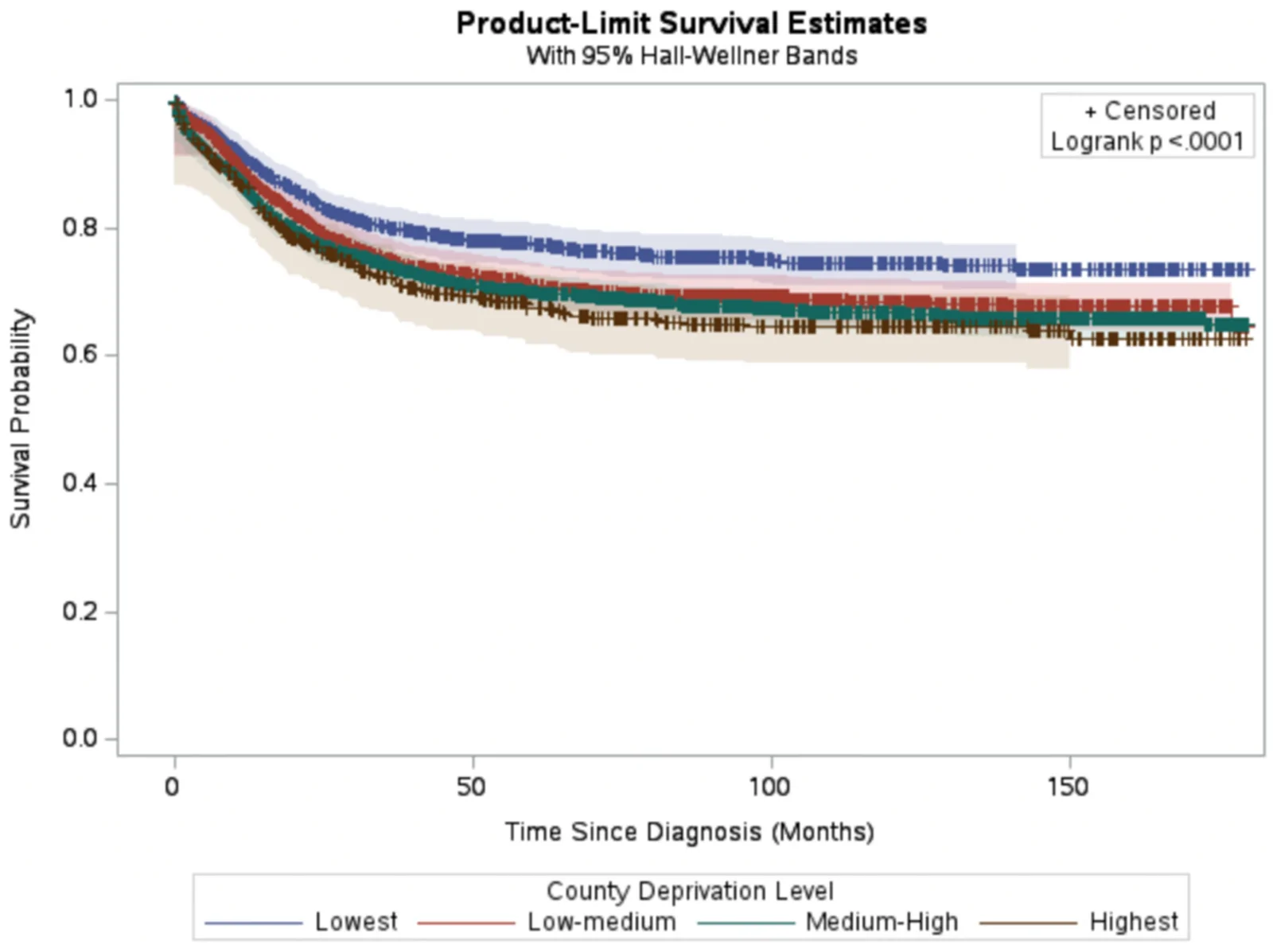
Survival Function by Socioeconomic Categorization of Counties Based on Deprivation Level.

**Figure 5:**
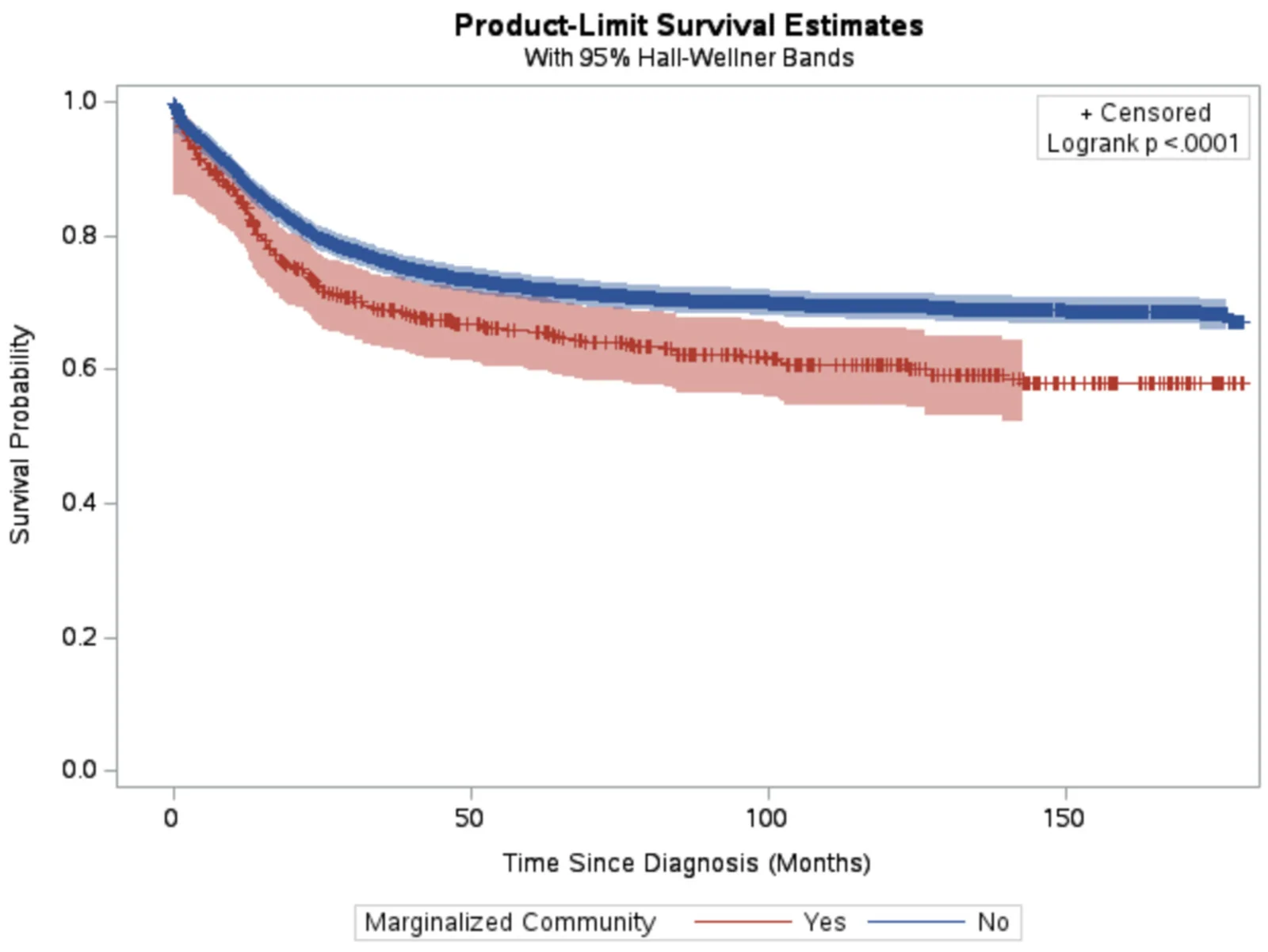
Survival Function by Community Marginalization Status.

Kaplan–Meier survival curves showed statistically significant differences in survival across the regional, county, and community-level of analysis (Figure 3, Figure 4 and Figure 5, Table 5).

At the regional level, log-rank testing found statistically significant survival differences across the four categories based on deprivation level (*p* = 0.0001). 1-, 3-, and 5-year survival estimates were estimated with results showing the lowest survival proportions in the most disadvantaged regions. Across the four strata, 5-year survival estimates were 78.26% (95% CI: 74.65–82.41) in the lowest, 71.80% (95% CI: 69.73–73.76) in low-medium, 69.81% (95% CI: 67.27–72.19) in medium-high, and 69.86% (95% CI: 67.49–72.10) in the highest category (Figure 1, Table 5).

At the county level, log-rank testing found statistically significant survival differences across the four categories based on deprivation level (*p* < 0.0001). 1-, 3-, and 5-year survival estimates were estimated with results showing the lowest survival proportions in the most disadvantaged regions. Across the four strata, 5-year survival estimates were 77.20% (95% CI: 74.60–79.58) in the lowest, 71.06% (95% CI: 68.68–73.29) in low-medium, 69.89% (95% CI: 67.95–71.74) in medium-high, and 67.47% (95% CI: 63.32–71.26) in the highest category (Figure 2, Table 5).

At the community level, survival was significantly lower in marginalized communities when estimating 1-, 3-, and 5-year survival. Log-rank testing found a statistically significant difference in survival outcomes comparing marginalized vs. non-marginalized communities (*p* < 0.0001). 5-year survival was 65.51% (95% CI: 61.33–69.35) among marginalized communities compared with 72.11% (95% CI: 70.82–73.35) among non-marginalized communities (Figure 3, Table 5).

### 3.4. Cox Proportional Hazards Models

Unadjusted and adjusted Cox proportional hazards models were performed to estimate the hazard ratios and 95% confidence intervals by regional, county, and community socioeconomic categorization of deprivation level (Table 6). In the regional and county analysis, the highest category was used as the reference. In the community-level marginalization analysis, ‘no,’ was used as the reference.

In unadjusted models, regional deprivation categorization is associated with survival outcomes (*p* = 0.0002) with patients residing in regions in the lowest category experience 32.9% better survival outcomes compared to patients residing in regions in the highest category. County socioeconomic categorization is also associated with survival outcomes in the unadjusted model (*p* < 0.0001). Patients residing in counties in the lowest category experience 34.00% better survival outcomes compared to patients residing in counties in the highest category. Overall, low-medium and medium-high categories were not significant compared to the reference category for both the regional and county analysis. Community marginalization status is significantly associated with lower survival in the unadjusted model (HR = 1.4, 95% CI: 1.20–1.60; *p* < 0.0001). Patients residing in marginalized areas have 38.7% worsened survival outcomes compared to patients residing in non-marginalized areas.

Adjusted models controlling for sex, age group, tumor behavior, geographical place of residence, and primary cancer type. The association between regional deprivation categorization remained significantly associated after adjustment (*p* < 0.0001). The lowest category exhibited significantly higher survival compared to the highest category (HR = 0.78, 95% CI: 0.74–0.95, *p* = 0.0143). Patients residing in regions in the lowest category experienced 22.3% better survival outcomes compared to patients residing in regions in the highest category. Overall county socioeconomic categorization is also significantly associated with survival outcomes in the adjusted model (*p* < 0.0001). The lowest county category exhibited significantly higher survival compared to the highest category (HR = 0.75, 95% CI: 0.62–0.91, *p* = 0.0143). Patients residing in counties in the lowest category experienced 25.1% better survival outcomes compared to patients residing in counties in the highest category. In both the regional and county adjusted analyses, low-medium and medium-high categories did not have significant survival outcomes compared to the reference category. After adjustment, community marginalization status is not significantly associated with survival outcomes (HR = 1.10, 95% CI: 0.94–1.28; *p* = 0.2255. This suggests that the unadjusted community marginalization-survival association is likely confounded by rurality.

### 3.5. Subgroup Analyses

#### 3.5.1. Effect of Marginalization Status in Rural Communities

**Figure 6:**
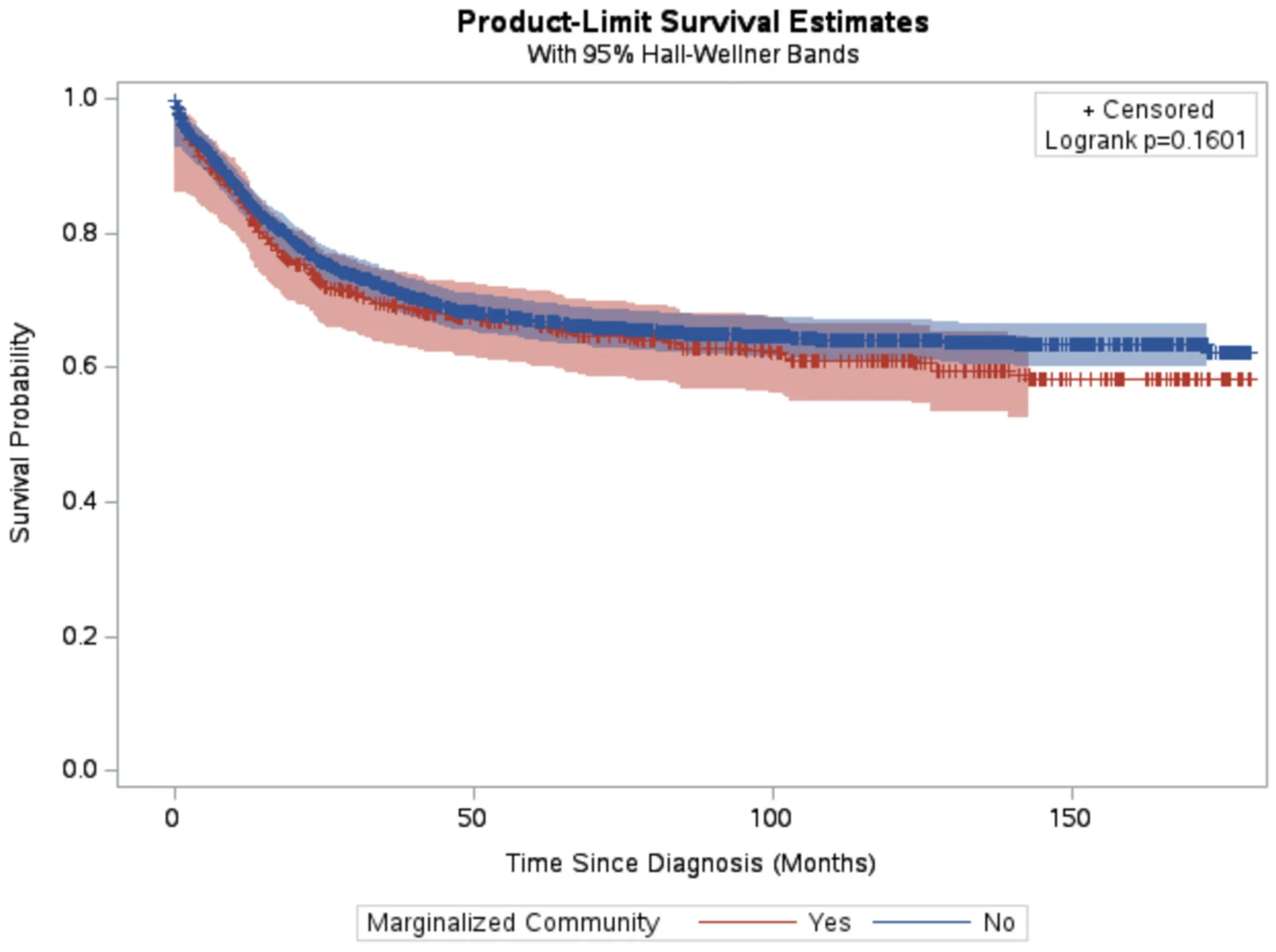
Survival Function by Community Marginalization Status Among Rural Pediatric Cancer Patients.

Among 2978 rural patients, Kaplan–Meier curves did not significantly differ by community marginalization level (*p* = 0.1601) (Figure 6; Table 7). 5-year survival was similar among rural residence in both marginalization and non-marginalized communities. 5-year survival for marginalized rural communities is 66.01% (95% CI: 61.80–69.87) and non-marginalized rural communities is 66.92% (95% CI: 64.85–68.90).

Cox regression analysis restricted to only rural patients was not statistically significant in unadjusted (HR = 1.17, 95% CI: 0.96–1.30, *p* = 0.1604) or adjusted models (HR = 1.10, 95% CI: 0.94–1.28, *p* = 0.2386) (Table 8). Among rural patients, cancer survival did not significantly vary across strata of community marginalization status.

#### 3.5.2. Effect of Rural Status

**Figure 7:**
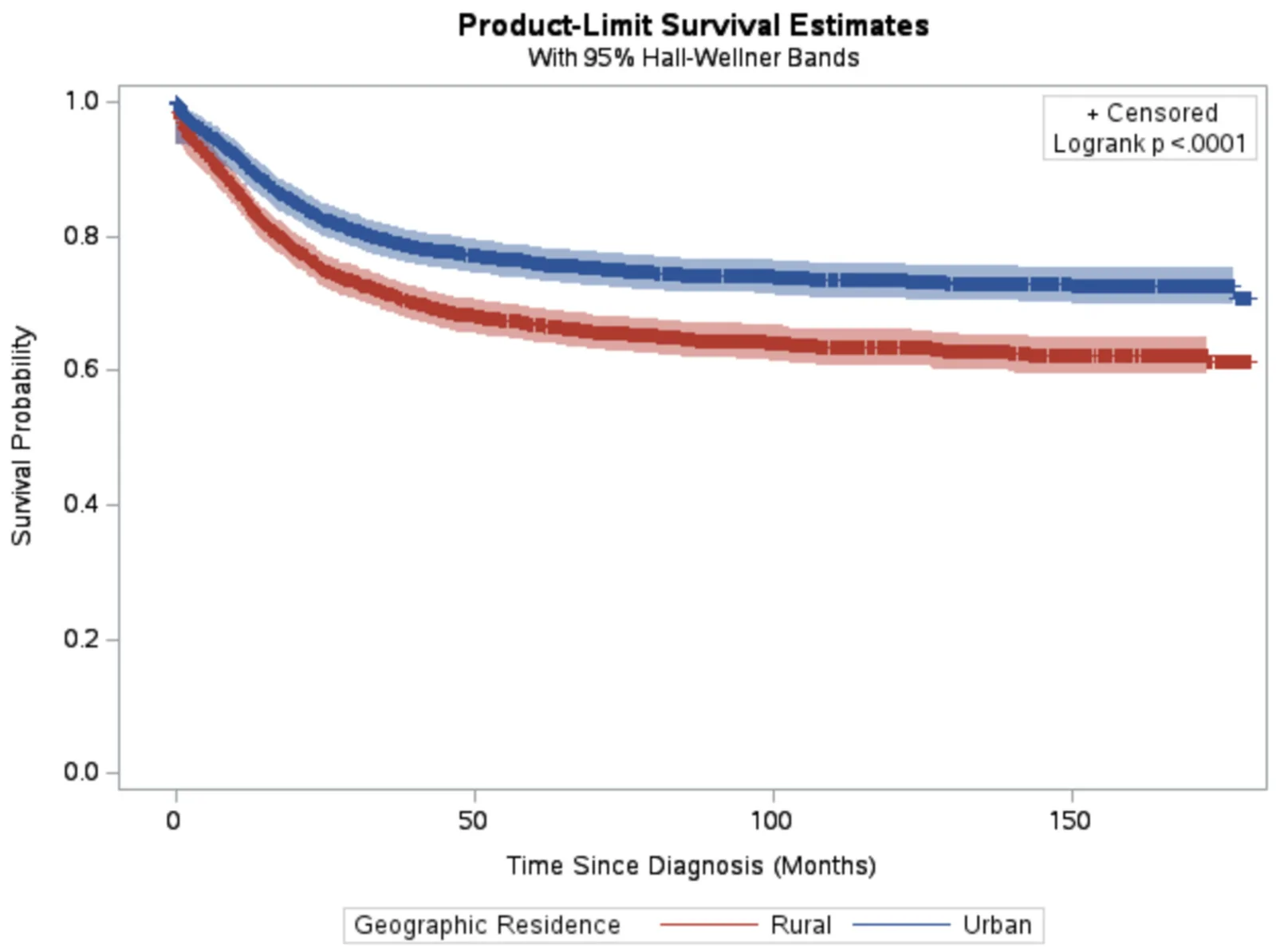
Survival Function by Urban/Rural Geographical Place of Residence Designation.

The second subgroup analysis estimated differences in survival by urban vs. rural geographical place of residence, independent of community marginalization status. Kaplan–Meier survival was significantly lower in rural communities when estimated 1-, 3-, and 5-year survival. Log-rank testing found a statistically significantly difference in survival outcomes comparing rural vs. urban communities (*p* < 0.0001) (Figure 7; Table 9). Survival probabilities were consistently lower for rural patients, with a 5-year survival of 66.76% (95% CI: 64.91, 68.53) compared to 75.93% (95% CI: 74.28, 77.48) among urban patients (Table 9).

In unadjusted Cox regression analysis, rural residence was associated with poorer survival outcomes compared to urban residence (HR =1.49, 95% CI: 1.35–1.64, *p* < 0.0001) (Table 10). Patients residing in rural communities experienced 49.1% worse survival outcomes compared to patients in urban communities. After adjusting for sex, age group, tumor behavior, and primary cancer type, rural residence remained significantly associated with pediatric survival outcomes (HR = 1.44, 95% CI: 1.30–1.58, *p* < 0.0001) (Table 10). Patients in rural communities have 43.6% worse survival outcomes compared to patients in urban communities. These findings suggest that rural residence is associated with pediatric cancer survival. Rurality is also correlated with broader socioeconomic factors, which may contribute to the observed disparities in the analysis.

## 4. Discussion

Our findings consistently support the assertion that survival rates are significantly influenced by area development and location, often described as a “postcode lottery” [73]. This is particularly significant for the urban-rural divide in Romania, as in other countries, regardless of overall economic wealth [8,11,24].

We observed significantly lower pediatric cancer survival among children in socioeconomically disadvantaged regions and counties, even after adjusting for several clinical and demographic factors. Area-level socioeconomic disparities in survival persist across administrative levels in Romania. At the community level (i.e., the smallest Territorial Administrative Unit), marginalization was associated with lower survival in unadjusted analyses but was no longer significant after adjustment. The association between community-level marginalization (multidimensional marginalization index value in the upper 20% of deprivation) and survival was not significant after adjustment. Community marginalization represents a multidimensional measure including poverty, education, housing conditions, infrastructure and utilities, and access to healthcare, with rurality representing one measure. Therefore, the observed differences in this analysis suggest that rurality may be an important component of the marginalization measure. In the analysis, most children living in marginalized communities also lived in rural areas, and the findings suggest that rural residence may be associated with poorer survival outcomes. Additionally, among patients residing in rural communities alone, marginalization was not associated with variation in survival outcomes Together, these findings suggest that rurality may explain the observed community-level differences in survival. The persistence of disparities across multiple geographic scales may reflect broader structural inequities in the healthcare system and the complexity of socioeconomic development factors, which may vary across different levels of granularity.

Our results are consistent with those reported in other registry-based analyses, which generally report higher mortality risks among children residing in socioeconomically disadvantaged neighborhoods [24,25,27,29]. In US-based studies, area-level deprivation measured using composite indices such as the Area Deprivation Index (ADI) has been associated with approximately 30–50% increased hazard of death when comparing the most deprived to the least deprived areas [11,12,13]. Trial-based analyses using individual-level poverty measures have similarly demonstrated increased relapse risk among children experiencing extreme household poverty during treatment [17]. Rural residence and greater geographic distance to pediatric oncology centers have also been associated with inferior survival across several cohorts [74,75]. However, not all analyses report uniform effects; some associations attenuate after adjustment for clinical variables or differ by cancer subtype, suggesting that socioeconomic gradients may be context- and diagnosis-specific [76].

Socioeconomic disparities influence survival through several interconnected pathways, including distance to tertiary care facilities providing comprehensive pediatric oncology services and the quality of locally available care. Specialized pediatric oncology services are currently available in only 7 of 42 counties. In our RNPOHR-based study population, fewer than 10% of cases were reported by more than one center, and over 80% of POH patients were managed exclusively in one of the 4 major university cities [39]. Given the geographic locations of the 4 cities hosting the major tertiary POH centers, most patients would likely need to travel 250–300 km for each visit related to treatment and follow-up care. The lack of pediatric services in nearby facilities that are adequately equipped for timely diagnosis and referral and capable of providing comprehensive supportive care is also likely to affect clinical outcomes that ultimately determine long-term, event-free survival for cancer patients [77].

A Eurostat report (2021) on quality of life in rural areas, examining access to main healthcare services, defined as the share of the population living in rural areas within 15 min driving time of a main healthcare service, ranked Romania at the lowest end of the EU, with less than 30% of the rural population able to reach a main HC service within 15 min of driving. Main healthcare services are generally considered to be hospitals offering inpatient services, or at least specialist services and tests (beyond the family doctor’s office capabilities) [78].

Another important factor is the affordability of care and the financial resilience at the family/household level, including the cost of disease coverage and household instability. Despite universal insurance coverage for all children in Romania, many families still face out-of-pocket expenses for a variety of cancer care-related items [79]. The high financial burden of treatment and the need for parents to accompany their sick child for months can lead to income losses and food, housing, or energy insecurity for families in low-SES territories, directly affecting a child’s overall health and ability to withstand aggressive therapy [74].

Children in high-poverty areas may have poorer baseline health or comorbidities due to poor nutrition or environmental exposures, increasing their susceptibility to severe treatment side effects. A national report on the health status of children and adolescents in Romania shows significant county-level differences in the incidence of iron deficiency anemia and malnutrition, with rates ranging from 20- to 25-fold between the lowest- and highest-incidence counties [80].

Across high-income settings, prior registry-based studies consistently demonstrate measurable survival gradients associated with socioeconomic disadvantage. In U.S. population-based cohorts, children residing in the most deprived neighborhoods—commonly defined using the Area Deprivation Index (ADI)—have shown approximately 30–50% higher mortality risk compared with those in the least deprived areas [11,12,13]. Rural residence has been associated with hazard ratios of approximately 1.2 to 1.5 for overall mortality, and greater distance to pediatric oncology centers has similarly been linked to significantly elevated mortality risk in children and adolescents [14,15,16]. In some analyses, socioeconomic disadvantage has also been associated with later stage at diagnosis.

European registry-based studies report comparable gradients. Nationwide analyses from Denmark, Germany, Greece, and the United Kingdom have documented statistically significant survival disadvantages among children from lower socioeconomic backgrounds, with excess mortality risks generally ranging from 10% to 40% depending on cancer type and the socioeconomic measure used [23,24,25,27,28]. Multi-country European comparisons further reveal persistent survival gaps between Western and Eastern Europe, with 5-year survival differences exceeding 10–15 percentage points in some cancer groups [4,5,66,67,72]. Systematic reviews and meta-analyses confirm that low socioeconomic status—whether defined at the individual or area level—is consistently associated with increased mortality risk across pediatric malignancies and geographic settings [22,81].

This study is based on a national, population-based pediatric cancer registry that includes validated cases from 2010 to 2024, enabling a comprehensive assessment of survival outcomes. By using multiple geographic levels, we simultaneously evaluated disparities at the regional, county, and community levels, an approach not previously investigated in Romania. The analysis adjusted for important clinical and demographic factors, including age, sex, primary cancer type, tumor behavior, and place of residence, thereby strengthening the validity of our findings. The study used epidemiological survival methods and incorporated subgroup analyses to better understand how rurality influences the association between community-level marginalization and survival.

Our study also has some limitations. Socioeconomic indicators were measured at the area level rather than the individual level, which may not fully capture household-level socioeconomic conditions. There was no standardized area deprivation index in Romania, so we used proxy indicators based on available data. While these indicators were selected for their relevance to pediatric cancer survival outcomes, this could limit comparability across studies. Some socioeconomic data were derived from census sources that may not fully align with the registry time period or study population. Lastly, although pediatric cancer is rare, the relatively small sample size and an estimated registry coverage of about 80% may lead to underreporting.

Overall, while the majority of international evidence supports the presence of socioeconomic survival gradients in pediatric oncology, findings are not universally consistent, The magnitude of these associations may depend on the selected deprivation measure, the geographic unit of analysis, the cancer type, and the health system context. Prior studies have generally assessed disparities at a single exposure level, without simultaneously evaluating socioeconomic disadvantage across multiple territorial strata within the same national registry framework. This adds complexity because area deprivation indicators and, more broadly, regional development indicators have changed over time in Romania, largely due to shifts in data-collection methodologies, the transition to market-based tracking, and the integration of European Union reporting standards. Since Romania has experienced a rapid decline in absolute poverty, methods for measuring relative poverty and socioeconomic segregation have been adapted, resulting in varied indicators over time. Area deprivation indicators in Romania have been subject to methodological shifts and differing institutional standards, leading to inconsistent interpretations of poverty over time. While the National Institute of Statistics (INS) and European bodies such as Eurostat provide standardized data, the criteria for defining “deprivation” have evolved to reflect changing socioeconomic standards.

Despite the heterogeneity of area deprivation measures, the resulting territorial socioeconomic configuration remained relatively stable over time, with the Bucharest-Ilfov, Center, and Western regions retaining their significant advantage over less affluent regions such as the North-East and Southern regions.

### Future Research

To address the interplay of multiple factors, a more in-depth exploration is required to identify and quantify critical determinants and to devise appropriate interventions.

A systematic approach would start with an analysis of the impact of geographic distance on survival outcomes and a clear understanding of the pediatric patient pathway throughout the cancer journey. The latter should assess the length and completion of critical milestones in the clinical process, such as delays in diagnosis and/or treatment initiation, compliance with and completion of the appropriate care plan, including protocols and follow-up schedules.

This approach should inform any optimization of the POH network and related services to ensure full geographic coverage, clarity of roles and competencies at every level of care, and improved resource utilization.

## 5. Conclusions

This is the first study in Romania that provides a comprehensive evaluation of geographic socioeconomic disparities in pediatric cancer survival. By assessing the relationship between socioeconomic deprivation and pediatric cancer survival at multiple geographical levels, the findings contribute to better understanding disparities throughout the country.

These findings have important implications for health policy strategies in Romania. Efforts to reduce disparities in pediatric cancer survival should prioritize improving access to appropriate proximity care, particularly in rural and socioeconomically disadvantaged areas. A strong network of pediatric cancer services, improved access to treatment, and equitable distribution of resources may help reduce survival inequities across a broader geographic context. Furthermore, addressing transportation barriers and improving access to diagnostic services, particularly in more remote areas, can facilitate earlier diagnosis and potentially eliminate gaps in follow-up. More broadly, reducing disparities will require addressing both underlying socioeconomic inequalities and barriers to accessing care that disproportionately affect underserved populations.

Future research should further explore the underlying mechanisms behind these disparities, including access to appropriate care, stage at diagnosis, treatment delays, and the intrinsic impact of socioeconomic constraints at the patient level. One could examine how travel distance from the place of residence to the treatment facility impacts survival outcomes. It is also important to determine whether disparities differ by cancer type, age group, or type of treatment. By identifying how disparities emerge, this study offers insights to guide targeted health policies and support stakeholders in improving pediatric cancer outcomes across Romania.

Not least, a productive reflection on pediatric cancer patient navigation within the healthcare system would emphasize the POH’s case management role within a highly diverse multidisciplinary team and incorporate lessons learned and tools developed by pioneering projects such as the POH outreach initiative and the shared-care model piloted by NGOs such as the Daruieste Aripi Association [82].

## Figures and Tables

**Table 1:** Patients’ Characteristics (n (%)).

	All Pediatric Cases (0–19 Years at Diagnosis) (n = 6247)	Children (0–14 Years at Diagnosis) (n = 4975)	Adolescents (15–19 Years at Diagnosis) (n = 1272)
**Age (y), mean ± SD**	**8.20 ± 5.81**	**6.09 ± 4.48**	**16.45 ± 1.29**
**Sex:**			
Male	**3499 (56.01)**	**2792 (56.12)**	**707 (55.58)**
Female	**2748 (43.99)**	**2183 (43.88)**	**565 (44.43)**
**Age categorization:**			
0–4	**2316 (37.07)**	**2316 (46.55)**	**0 (0.00)**
5–9	**1289 (20.63)**	**1289 (25.91)**	**0 (0.00)**
10–14	**1370 (21.93)**	**1370 (27.54)**	**0 (0.00)**
15+	**1272 (20.36)**	**0 (0.00)**	**1272 (100.00)**
**Death:**			
Yes	**1748 (27.98)**	**1356 (27.26)**	**392 (30.82)**
No	**4499 (72.02)**	**3619 (72.74)**	**880 (69.18)**
**Economic Development Region:**			
North-East	**1231 (19.71)**	**954 (19.18)**	**277 (21.78)**
Bucharest-Ilfov	**696 (11.14)**	**593 (11.92)**	**103 (8.10)**
Center	**795 (12.73)**	**645 (12.96)**	**150 (11.79)**
North-West	**975 (15.61)**	**747 (15.02)**	**228 (17.92)**
South-East	**731 (11.70)**	**593 (11.92)**	**138 (10.85)**
South-Mutenia	**848 (13.57)**	**687 (13.81)**	**161 (12.66)**
South-West Oltenia	**534 (8.55)**	**428 (8.60)**	**106 (8.33)**
West	**437 (7.00)**	**328 (6.59)**	**109 (8.57)**
**Socioeconomic Deprivation (Regional Level):**			
Lowest	**696 (11.14)**	**593 (11.92)**	**103 (8.10)**
Low-medium	**2207 (35.33)**	**1720 (34.57)**	**487 (38.29)**
Medium-high	**1579 (25.28)**	**1280 (25.73)**	**299 (23.51)**
Highest	**1765 (28.25)**	**1382 (27.78)**	**383 (30.22)**
**Socioeconomic Deprivation (County Level):**			
Lowest	**1299 (20.79)**	**1086 (21.83)**	**213 (16.75)**
Low-medium	**1710 (27.37)**	**1341 (26.95)**	**369 (29.01)**
Medium-high	**2616 (41.88)**	**2057 (41.35)**	**559 (43.95)**
Highest	**622 (9.96)**	**491 (9.87)**	**131 (10.30)**
**Geographical Place of Residence:**			
Urban	**3213 (51.43)**	**2600 (52.26)**	**613 (48.19)**
Rural	**3023 (48.39)**	**2367 (47.58)**	**656 (51.57)**
**Residence in a Most Marginalized Community:**			
Yes	**598 (9.57)**	**411 (8.26)**	**187 (14.70)**
No	**5649 (90.43)**	**4564 (91.74)**	**1085 (85.30)**
**Behavior:**			
Malignant	**6055 (96.93)**	**4804 (96.56)**	**1251 (98.35)**
Benign	**11 (0.18)**	**10 (0.20)**	**1 (0.08)**
Borderline, in situ	**181 (2.90)**	**161 (3.24)**	**20 (1.57)**
**Primary Cancer Type:**			
I: Leukemias, myeloproliferative diseases and myelodysplastic diseases	**1883 (30.14)**	**1661 (33.79)**	**202 (15.88)**
II: Lymphomas and reticuloendothelial neoplasms	**965 (15.45)**	**678 (13.63)**	**287 (22.56)**
III: CNS and miscellaneous intracranial and intraspinal neoplasms	**911 (14.58)**	**802 (16.12)**	**109 (8.57)**
IV: Neuroblastoma and other peripheral nervous cell tumors	**379 (6.07)**	**376 (7.56)**	**3 (0.24)**
V: Retinoblastoma	**107 (1.71)**	**106 (2.13)**	**1 (0.08)**
VI: Renal tumors	**318 (5.09)**	**311 (6.25)**	**7 (0.55)**
VII: Hepatic tumors	**82 (1.31)**	**75 (1.51)**	**7 (0.55)**
VIII: Malignant bone tumors	**434 (6.95)**	**288 (5.79)**	**146 (11.48)**
IX: Soft tissue bone and other extraosseous sarcomas	**462 (7.40)**	**327 (6.57)**	**135 (10.61)**
X: Germ cell tumors, trophoblastic tumors, and neoplasms of gonads	**319 (5.11)**	**164 (3.30)**	**155 (12.19)**
XI: other malignant epithelial neoplasms and malignant melanomas	**362 (5.79)**	**147 (2.95)**	**215 (16.90)**
XII: other and unspecified malignant neoplasms	**25 (0.40)**	**20 (0.40)**	**5 (0.39)**
**Treatment Type (Unimodal):**			
No treatment	**126 (2.02)**	**102 (2.05)**	**24 (1.89)**
Surgery	**2108 (33.74)**	**1635 (32.86)**	**473 (37.19)**
Radiotherapy	**663 (10.61)**	**506 (10.17)**	**157 (12.34)**
Systemic Therapy	**4890 (78.28)**	**4035 (81.11)**	**855 (67.22)**
**Treatment Type (Multimodal (total)):**			
Occurrence of surgery in combination	**2013 (32.22)**	**1656 (33.29)**	**356 (27.99)**
Occurrence of radiotherapy in combination	**1008 (16.13)**	**802 (16.12)**	**206 (16.19)**
Occurrence of systemic therapy in combination	**2197 (35.17)**	**1779 (35.76)**	**418 (32.86)**

**Table 2:** Characteristics of Socioeconomic Categorization of Regions Based on Deprivation Level (n (%)).

Variable	Lowest (n = 696)	Low-Medium (n = 2207)	Medium-High (n = 1579)	Highest (n = 1765)	*p*-Value
**Age (y), mean ± SD**	**7.21 ± 5.32**	**8.37 ± 5.91**	**8.06 ± 5.67**	**8.50 ± 5.86**	***p* < 0.0001**
**Age group**					**0.0002**
0–4	**305 (43.82)**	**802 (36.34)**	**584 (36.99)**	**625 (35.41)**	
5–9	**141 (20.26)**	**459 (20.80)**	**344 (21.79)**	**345 (19.55)**	
10–14	**147 (21.12)**	**459 (20.80)**	**352 (22.29)**	**412 (23.34)**	
15–19	**103 (22.07)**	**487 (22.07)**	**299 (18.94)**	**383 (21.70)**	
**Sex**					**0.7678**
Male	**378 (54.31)**	**1233 (55.87)**	**889 (56.30)**	**999 (56.60)**	
Female	**318 (45.69)**	**974 (44.13)**	**690 (43.70)**	**766 (43.40)**	
**Geographical Place of Residence:**					***p* < 0.0001**
Urban	**615 (88.36)**	**1149 (52.06)**	**710 (44.97)**	**739 (41.87)**	
Rural	**81 (11.64)**	**1056 (47.85)**	**867 (54.91)**	**1019 (57.73)**	
**Behavior**					**0.0210**
Malignant	**671 (96.41)**	**2146 (97.24)**	**1514 (95.88)**	**1724 (97.68)**	
Benign	**0 (0.00)**	**5 (0.23)**	**2 (0.13)**	**4 (0.23)**	
Borderline, in situ	**25 (3.59)**	**56 (2.54)**	**63 (3.99)**	**37 (2.10)**	
**Primary Cancer Type**					***p* < 0.0001**
I: Leukemias, myeloproliferative diseases and myelodysplastic diseases	**232 (33.33)**	**643 (29.13)**	**475 (30.08)**	**533 (30.20)**	
II: Lymphomas and reticuloendothelial neoplasms	**91 (13.07)**	**343 (15.54)**	**238 (15.07)**	**293 (16.60)**	
III: CNS and miscellaneous intracranial and intraspinal neoplasms	**114 (16.38)**	**289 (13.09)**	**255 (16.15)**	**253 (14.33)**	
IV: Neuroblastoma and other peripheral nervous cell tumors	**51 (7.33)**	**144 (6.52)**	**82 (5.19)**	**102 (5.78)**	
V: Retinoblastoma	**13 (1.87)**	**34 (1.54)**	**27 (1.71)**	**33 (1.87)**	
VI: Renal tumors	**41 (5.89)**	**109 (4.94)**	**87 (5.51)**	**81 (4.59)**	
VII: Hepatic tumors	**9 (1.29)**	**34 (1.54)**	**24 (1.52)**	**15 (0.85)**	
VIII: Malignant bone tumors	**42 (6.03)**	**144 (6.52)**	**134 (8.49)**	**114 (6.46)**	
IX: Soft tissue bone and other extraosseous sarcomas	**52 (7.47)**	**168 (7.61)**	**116 (7.35)**	**126 (7.14)**	
X: Germ cell tumors, trophoblastic tumors, and neoplasms of gonads	**29 (4.17)**	**129 (5.85)**	**67 (4.24)**	**94 (5.33)**	
XI: other malignant epithelial neoplasms and malignant melanomas	**21 (3.02)**	**164 (7.43)**	**69 (4.37)**	**108 (6.12)**	
XII: other and unspecified malignant neoplasms	**1 (0.14)**	**6 (0.27)**	**5 (0.32)**	**13 (0.74)**	

**Table 3:** Characteristics of Socioeconomic Categorization of Counties Based on Deprivation Level: (n (%)).

Variable	Lowest (n = 1299)	Low-Medium (n = 1710)	Medium-High (n = 2616)	Highest (n = 622)	*p*-Value
**Age (y), mean ± SD**	**7.45 ± 5.64**	**8.41 ± 5.87**	**8.32 ± 5.85**	**8.64 ± 5.69**	***p* < 0.0001**
**Age group**					**0.0002**
0–4	**549 (42.26)**	**612 (35.79)**	**956 (36.54)**	**199 (31.99)**	
5–9	**270 (20.79)**	**351 (20.53)**	**524 (20.03)**	**144 (23.15)**	
10–14	**267 (20.55)**	**378 (22.11)**	**577 (22.06)**	**148 (23.79)**	
15–19	**213 (16.40)**	**369 (21.58)**	**559 (21.37)**	**131 (21.06)**	
**Sex**					**0.8602**
Male	**714 (54.97)**	**962 (56.26)**	**1471 (56.23)**	**352 (56.59)**	
Female	**585 (45.03)**	**748 (43.74)**	**1145 (43.77)**	**270 (43.41)**	
**Geographical Place of Residence:**					***p* < 0.0001**
Urban	**980 (75.44)**	**881 (51.52)**	**1113 (42.55)**	**239 (38.42)**	
Rural	**319 (24.56)**	**827 (48.36)**	**1496 (57.19)**	**381 (61.25)**	
**Behavior**					***p* < 0.0001**
Malignant	**1261 (97.07)**	**1660 (97.08)**	**2538 (97.02)**	**596 (95.82)**	
Benign	**1 (0.08)**	**4 (0.23)**	**5 (0.19)**	**1 (0.16)**	
Borderline, in situ	**37 (2.85)**	**46 (2.69)**	**73 (2.79)**	**25 (4.02)**	
**Primary Cancer Type**					**0.0212**
I: Leukemias, myeloproliferative diseases and myelodysplastic diseases	**416 (32.02)**	**495 (28.95)**	**762 (29.13)**	**210 (33.76)**	
II: Lymphomas and reticuloendothelial neoplasms	**177 (13.63)**	**271 (15.85)**	**429 (16.40)**	**88 (14.15)**	
III: CNS and miscellaneous intracranial and intraspinal neoplasms	**189 (14.55)**	**250 (14.62)**	**379 (14.49)**	**93 (14.95)**	
IV: Neuroblastoma and other peripheral nervous cell tumors	**105 (8.08)**	**99 (5.79)**	**145 (5.54)**	**30 (4.82)**	
V: Retinoblastoma	**25 (1.92)**	**19 (1.11)**	**55 (2.10)**	**8 (1.29)**	
VI: Renal tumors	**65 (5.00)**	**93 (5.44)**	**128 (4.89)**	**32 (5.14)**	
VII: Hepatic tumors	**17 (1.31)**	**25 (1.92)**	**34 (1.30)**	**6 (0.96)**	
VIII: Malignant bone tumors	**79 (6.08)**	**120 (7.02)**	**188 (7.19)**	**47 (7.56)**	
IX: Soft tissue bone and other extraosseous sarcomas	**99 (7.62)**	**121 (7.08))**	**191 (7.30)**	**51 (8.20)**	
X: Germ cell tumors, trophoblastic tumors, and neoplasms of gonads	**69 (5.31)**	**101 (5.91)**	**127 (4.85)**	**22 (3.54)**	
XI: other malignant epithelial neoplasms and malignant melanomas	**56 (4.31)**	**109 (6.37)**	**163 (6.23)**	**34 (5.47)**	
XII: other and unspecified malignant neoplasms	**2 (0.15)**	**7 (0.41)**	**15 (0.57)**	**1 (0.16)**	

**Table 4:** Characteristics by Community Marginalization Status (n (%)).

Variable	Yes (n = 598)	No (n = 5638)	*p*-Value
**Age (y), mean ± SD**	**9.74 ± 5.91**	**8.03 ± 5.77**	***p* < 0.0001**
**Age group**			***p* < 0.0001**
0–4	**164 (27.42)**	**2150 (38.13)**	
5–9	**115 (19.23)**	**1169 (20.73)**	
10–14	**132 (22.07)**	**1237 (21.94)**	
15–19	**187 (31.27)**	**1082 (19.19)**	
**Sex**			**0.1181**
Male	**353 (59.03)**	**3140 (55.69)**	
Female	**245 (40.97)**	**2498 (44.31)**	
**Geographical Place of Residence:**			***p* < 0.0001**
Urban	**14 (2.34)**	**3199 (56.74)**	
Rural	**584 (97.66)**	**2439 (43.26)**	
**Behavior**			***p* < 0.0001**
Malignant	**575 (96.15)**	**5469 (97.00)**	
Benign	**0 (0.00)**	**11 (0.20)**	
Borderline, in situ	**23 (3.85)**	**158 (2.80)**	
**Primary Cancer Type**			**0.5081**
I: Leukemias, myeloproliferative diseases and myelodysplastic diseases	**163 (27.26)**	**1718 (30.47)**	
II: Lymphomas and reticuloendothelial neoplasms	**98 (16.39)**	**864 (15.32)**	
III: CNS and miscellaneous intracranial and intraspinal neoplasms	**103 (17.22)**	**808 (14.33)**	
IV: Neuroblastoma and other peripheral nervous cell tumors	**29 (4.85)**	**349 (6.19)**	
V: Retinoblastoma	**10 (1.67)**	**97 (1.72)**	
VI: Renal tumors	**24 (4.01)**	**294 (5.21)**	
VII: Hepatic tumors	**7 (1.17)**	**75 (1.33)**	
VIII: Malignant bone tumors	**45 (7.53)**	**389 (6.90)**	
IX: Soft tissue bone and other extraosseous sarcomas	**48 (8.03)**	**413 (7.33)**	
X: Germ cell tumors, trophoblastic tumors, and neoplasms of gonads	**29 (4.85)**	**287 (5.09)**	
XI: other malignant epithelial neoplasms and malignant melanomas	**40 (6.69)**	**322 (5.71)**	
XII: other and unspecified malignant neoplasms	**2 (0.33)**	**22 (0.39)**	

**Table 5:** Kaplan–Meier Estimated 1-, 3- and 5-Year Survival Among Pediatric Cancer Patients by Regional, County, and Community-level Socioeconomic Categorization of Deprivation Level.

Analysis Level	Number of Patients	Number of Deaths	1-Year Survival (95% CI)	3-Year Survival (95% CI)	5-Year Survival (95% CI)	Log-Rank *p*-Value
**Region**						***p* = 0.0001**
Lowest	**686**	**138**	**91.97 (89.62, 93.81)**	**81.06** **(77.69, 83.97)**	**78.26** **(74.65, 81.41)**	
Low-medium	**2193**	**591**	**87.80** **(86.33, 89.11)**	**75.63** **(73.69, 77.45)**	**71.80** **(69.73, 73.76)**	
Medium-high	**1556**	**456**	**87.91** **(86.16, 89.46)**	**73.76** **(71.38, 75.98)**	**69.81** **(67.27, 72.19)**	
Highest	**1715**	**502**	**85.53** **(83.75, 87.12)**	**73.88** **(71.65, 75.97)**	**69.86** **(67.49, 72.10)**	
**County**						***p* < 0.0001**
Lowest	**1285**	**275**	**90.71** **(88.95, 92.20)**	**80.07** **(77.64, 82.27)**	**77.20** **(74.60, 79.58)**	
Low-medium	**1698**	**471**	**88.23** **(86.58, 89.69)**	**74.92** **(72.68, 77.00)**	**71.06** **(68.68, 73.29)**	
Medium-high	**2557**	**749**	**86.12** **(84.70, 87.42)**	**73.84** **(72.02, 75.57)**	**69.89** **(67.95, 71.74)**	
Highest	**610**	**192**	**86.10** **(83.05, 88.63)**	**72.36** **(68.46, 75.85)**	**67.47** **(63.32, 71.26)**	
**Most Marginalized Community**						***p* < 0.0001**
Yes	**594**	**213**	**84.61** **(81.41, 87.29)**	**68.80** **(64.77, 72.46)**	**65.51** **(61.33, 69.35)**	
No	**5545**	**1471**	**88.00** **(87.10, 88.84)**	**75.98** **(74.78, 77.14)**	**72.11** **(70.82, 73.35)**	

**Table 6:** Unadjusted/Adjusted Hazard Ratios (95% CI) by Regional, County, and Community-level Socioeconomic Categorization of Deprivation Level Among Pediatric Cancer Patients.

Analysis Level	Unadjusted Model	*p*-Value	Adjusted Model	*p*-Value
**Region**		***p* = 0.0002**		***p* < 0.0001**
Lowest	**0.67** **(0.56, 0.81)**	***p* < 0.0001**	**0.78** **(0.74, 0.96)**	***p* = 0.0143**
Low-medium	**0.90** **(0.80, 1.02)**	***p* = 0.0937**	**0.95** **(0.85, 1.078)**	***p* = 0.4369**
Medium-high	**1.00** **(0.88, 1.13)**	***p* = 0.9812**	**0.99** **(0.87, 1.12)**	***p* = 0.8453**
Highest	**1.00 (reference)**		**1.00 (reference)**	
**County**		***p* < 0.0001**		***p* < 0.0001**
Lowest	**0.66** **(0.55, 0.79)**	***p* < 0.0001**	**0.75** **(0.62, 0.91)**	***p* = 0.0028**
Low-medium	**0.85** **(0.72, 1.00)**	***p* = 0.0555**	**0.89** **(0.75, 1.05)**	***p* = 0.1586**
Medium-high	**0.92** **(0.79, 1.08)**	***p* = 0.3210**	**0.94** **(0.80, 1.10)**	***p* = 0.4209**
Highest	**1.00 (reference)**		**1.00 (reference)**	
**Marginalized Community**		***p* < 0.0001**		***p* = 0.2255**
Yes	**1.39** **(1.20, 1.60)**	***p* < 0.0001**	**1.10** **(0.94, 1.28)**	***p* = 0.2255**
No	**1.00 (reference)**		**1.00 (reference)**	

**Table 7:** Kaplan–Meier Estimated 1-, 3- and 5-Year Survival Among Rural Pediatric Cancer Patients by Community Marginalization Status.

Marginalized Community	Number of Patients	Number of Deaths	1-Year Survival (95% CI)	3-Year Survival (95% CI)	5-Year Survival (95% CI)	Log-Rank *p*-Value
Yes	**580**	**206**	**84.24** **(80.98, 86.99)**	**69.17** **(65.11, 72.86)**	**66.01** **(61.80, 69.87)**	***p* = 0.1601**
No	**2398**	**753**	**85.10** **(83.59, 86.48)**	**71.68** **(69.74, 73.51)**	**66.92** **(64.85, 68.90)**	

**Table 8:** Unadjusted/Adjusted Hazard Ratios (95% CI) Among Rural Pediatric Cancer Patients by Community Marginalization Status.

Marginalized Community	Unadjusted Model	*p*-Value	Adjusted Model	*p*-Value
Yes	**1.12** **(0.96, 1.30)**	***p* = 0.1604**	**1.10** **(0.94, 1.28)**	***p* = 0.2336**
No	**1.00** **(reference)**		**1.00** **(reference)**	

**Table 9:** Kaplan–Meier Estimated 1-, 3- and 5-Year Survival by Urban/Rural Geographical Place of Residence Designation.

Geographical Place of Residence	Number of Patients	Number of Deaths	1-Year Survival (95% CI)	3-Year Survival (95% CI)	5-Year Survival (95% CI)	Log-Rank *p*-Value
Urban	**3161**	**725**	**90.26** **(89.15, 91.26)**	**79.17** **(77.63, 80.61)**	**75.93** **(74.28, 77.48)**	***p* < 0.0001**
Rural	**2978**	**959**	**84.93** **(83.58, 86.18)**	**71.19** **(69.45, 72.85)**	**66.76** **(64.91, 68.53)**	

**Table 10:** Unadjusted/Adjusted Hazard Ratios (95% CI) by Urban/Rural Geographical Place of Residence Designation.

Geographical Place of Residence	Unadjusted Model	*p*-Value	Adjusted Model	*p*-Value
Urban	**1.00** **(reference)**		**1.00** **(reference)**	
Rural	**1.49** **(1.35, 1.64)**	***p* < 0.0001**	**1.44** **(1.30, 1.58)**	***p* < 0.0001**

## Data Availability

The raw data supporting the conclusions of this article will be made available by the authors, without undue reservation.

## Supplementary Materials

The following supporting information can be downloaded at: https://www.mdpi.com/article/10.3390/cancers18162627/s1, Figure S1: Age-stratified Survival Function by Socioeconomic Categorization of Regions Based on Deprivation Level. Figure S2: Age-stratified Survival Function by Socioeconomic Categorization of Counties Based on Deprivation Level. Figure S3: Age-stratified Survival Function by Community Marginalization Status. Table S1: Age-stratified Kaplan-Meier Estimated 1-, 3-, and 5-Year Survival Among Pediatric Cancer Patients by Regional, County, and Community-level Socioeconomic Categorization of Deprivation Level. Table S2: Age-stratified Unadjusted/Adjusted Hazard Ratios (95% CI) by Regional, County, and Community-level Socioeconomic Categorization of Deprivation Level Among Pediatric Cancer Patients.
