# Supplementary material for: Disparities in Area Socioeconomic Development and Pediatric Cancer Survival in Romania—A National Pediatric Registry Study on Multiple Geographic Levels
Source: Cancers (Basel). 2026 Aug 14;18(16):2627. doi: 10.3390/cancers18162627 (PMC13511483; doi:10.3390/cancers18162627)

Supplemental Materials

Age-stratified sensitivity analyses were performed among patients aged 0-14 years and 15-19 years to evaluate whether the associations observed in the main text differed by age. Cox proportional hazard regression models and Kaplan-Meier survival curves were repeated for each age group at the regional, county, and community levels using the same analytical approach as the primary analyses. Adjusted Cox proportional hazard models included the same covariates as the primary analyses.

Table S1. Age-stratified Kaplan-Meier Estimated 1-, 3-, and 5-Year Survival Among Pediatric Cancer Patients by Regional, County, and Community-level Socioeconomic Categorization of Deprivation Level.

Analysis Level	Patients aged 0-14 years (N = 4,898)						Patients aged 15-19 years (N = 1,252)					
	Number of Patients	Number of deaths	1-year survival (95% CI)	3-year survival (95% CI)	5-year survival (95% CI)	Log-rank p-value	Number of Patients	Number of Deaths	1-year survival (95% CI)	3-year survival (95% CI)	5-year survival (95% CI)	Log-rank p-value
**Region**												
Lowest	**585**	**109**	**92.06 (89.86, 94.32)**	**82.68 (79.50, 85.99)**	**80.19 (76.77, 83.76)**	**p=0.0002**	**101**	**29**	**91.29 (85.68, 97.26)**	**70.87 (61.61, 81.52)**	**66.12 (56.31, 77.64)**	**p=0.1857**
Low-medium	**1,711**	**459**	**87.65 (86.08, 89.24)**	**75.73 (73.64, 77.89)**	**71.48 (69.21, 73.81)**		**482**	**132**	**88.32 (85.46, 91.27)**	**75.29 (71.38, 79.41)**	**72.88 (68.81, 77.18)**	
Medium-high	**1,262**	**361**	**87.61 (85.78, 89.47)**	**73.84 (71.34, 76.44)**	**70.75 (68.12, 73.49)**		**294**	**95**	**89.23 (85.65, 92.95)**	**73.42 (68.24, 78.99)**	**65.75 (60.02, 72.02)**	
Highest	**1,340**	**376**	**85.76 (83.89, 87.68)**	**74.79 (72.41, 77.24)**	**70.93 (68.40, 73.55)**		**375**	**126**	**84.66 (81.04, 88.45)**	**70.67 (66.03, 75.63)**	**66.07 (61.17, 71.37)**	
**County**												
Lowest	**1,074**	**219**	**90.94 (89.21, 92.71)**	**81.24 (78.82. 83.74)**	**78.35 (75.73, 81.06)**	**p<0.0001**	**211**	**56**	**89.51 (85.36, 93.87)**	**73.93 (67.79, 80.62)**	**71.19 (64.79, 78.23)**	**p=0.4581**
Low-medium	**1,336**	**364**	**87.90 (86.35, 89.69)**	**75.05 (72.66, 77.51)**	**71.04 (68.49, 73.69)**		**362**	**107**	**89.46 (86.31, 92.73)**	**74.44 (69.87, 79.32)**	**71.11 (66.29, 76.28)**	
Medium-high	**2,008**	**574**	**85.88 (84.38, 87.43)**	**74.37 (72.41, 76.39)**	**70.60 (68.51, 72.76)**		**549**	**175**	**87.02 (84.21, 89.93)**	**71.91 (68.09, 75.95)**	**67.30 (63.24, 71.62)**	
Highest	**480**	**148**					**130**	**44**	**82.49**	**73.52**	**64.28**	

			**87.05 (84.07, 90.14)**	**72.06 (68.00, 76.35)**	**68.39 (64.12, 72.95)**				**(76.09, 89.43)**	**(66.00, 81.90)**	**(55.91, 73.89)**	
**Most Marginalized Community**												
Yes	**4,490**	**1,169**	**87.91**	**76.31**	**72.63 (71.24, 74.04)**	**p=0.0025**	**1,066**	**305**	**88.29 (86.34, 90.28)**	**74.56 (71.85, 77.38)**	**69.91 (66.99, 72.96)**	**p=0.0024**
No	**408**	**136**	**84.80**	**70.37**	**67.08 (62.43, 72.07)**		**186**	**77**	**84.22 (79.11, 89.66)**	**65.45 (58.82, 72.82)**	**62.17 (55.37, 69.81)**	

Table S2. Age-stratified Unadjusted/Adjusted Hazard Ratios (95% CI) by Regional, County, and Community-level Socioeconomic Categorization of Deprivation Level Among Pediatric Cancer Patients.

**Analysis Level**	**Patients aged 0-14 years (N = 4,898)**				**Patients aged 15-19 years (N = 1,252)**			
	**Unadjusted Model**	**p-value**	**Adjusted Model**	**p-value**	**Unadjusted Model**	**p-value**	**Adjusted Model**	**p-value**
**Region**		**p=0.0002**		**p<0.0001**		**p=0.1876**		**p=0.5899**
Lowest	**0.64 (0.52, 0.79)**	**p<0.0001**	**0.75 (0.61, 0.94)**	**p=0.0120**	**0.91 (0.61, 1.36)**	**0.6327**	**1.00 (0.66, 1.52)**	**p=0.9943**
Low-medium	**0.95 (0.82, 1.08)**	**p=0.4243**	**0.98 (0.85, 1.13)**	**p=0.7682**	**0.77 (0.60, 0.98)**	**0.0364**	**0.86 (0.67, 1.11)**	**p=0.2463**
Medium-high	**1.02 (0.88, 1.17)**	**p=0.7834**	**1.03 (0.89, 1.19)**	**p=0.6686**	**0.95 (0.72, 1.23)**	**0.6782**	**0.87 (0.66, 1.14)**	**p=0.3020**
Highest	**1.00 (reference)**		**1.00 (reference)**		**1.00 (reference)**		**1.00 (reference)**	
**County**		**p<0.0001**		**p<0.0001**		**p=0.4594**		**p=0.8631**
Lowest	**0.64 (0.55, 0.79)**	**p<0.0001**	**0.70 (0.57, 0.87)**	**p=0.0013**	**0.78 (0.52, 1.15)**	**p=0.2135**	**1.03 (0.69, 1.55)**	**p=0.8810**
Low-medium	**0.86 (0.71, 1.04)**	**p=0.1225**	**0.87 (0.72, 1.05)**	**p=0.1564**	**0.80 (0.57, 1.14)**	**p=0.2232**	**0.98 (0.68, 1.40)**	**p=0.9015**
Medium-high	**0.93 (0.77, 1.11)**	**p=0.3998**	**0.93 (0.77, 1.11)**	**p=0.4020**	**0.91 (0.65, 1.27)**	**p=0.5815**	**1.08 (0.78, 1.51)**	**p=0.6366**
Highest	**1.00 (reference)**		**1.00 (reference)**		**1.00 (reference)**		**1.00 (reference)**	

**Marginalized Community**		**p=0.0024**		**p=0.6930**		**p=0.0026**		**p=0.0478**
Yes	**1.32 (1.10, 1.57)**	**p=0.0024**	**1.04 (0.86, 1.25)**	**p=0.6930**	**1.47 (1.14, 1.89)**	**p=0.0026**	**1.32 (1.00, 1.75)**	**p=0.0478**
No	**1.00 (reference)**		**1.00 (reference)**		**1.00 (reference)**		**1.00 (reference)**	

Figure S1. Age-stratified Survival Function by Socioeconomic Categorization of Regions Based on Deprivation Level.

Figure S1a: Patients aged 0-14 years

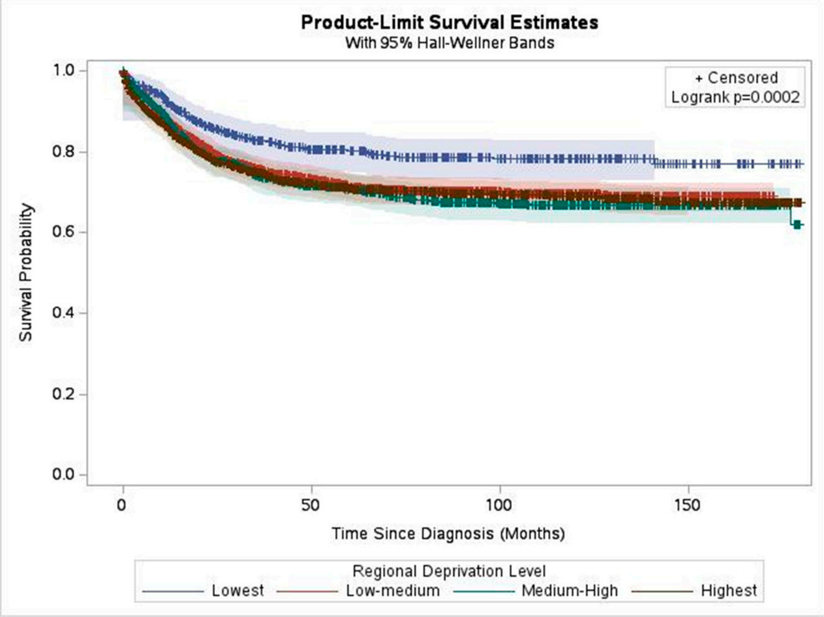

Figure S1b: Patients aged 15-19 years

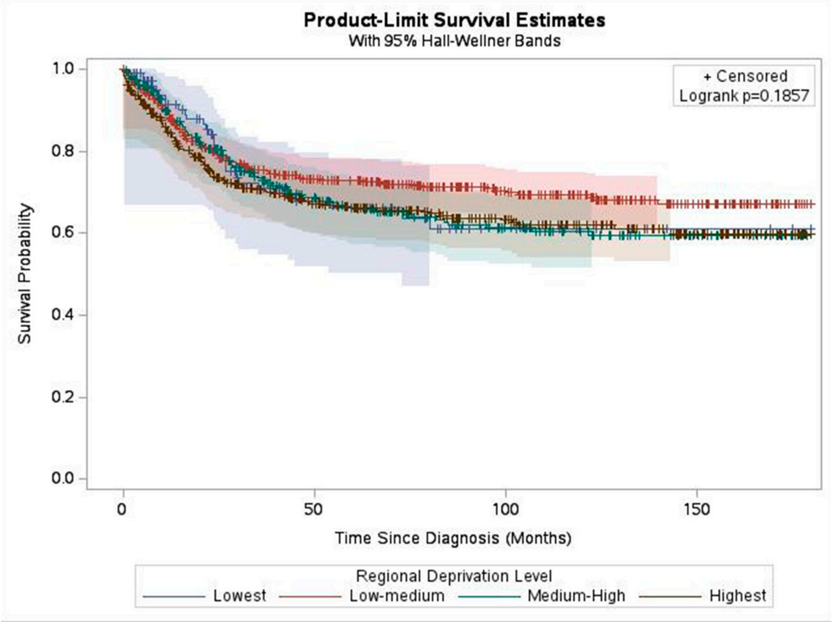

Figure S2. Age-stratified Survival Function by Socioeconomic Categorization of Counties Based on Deprivation Level.

Figure S2a: Patients aged 0-14 years

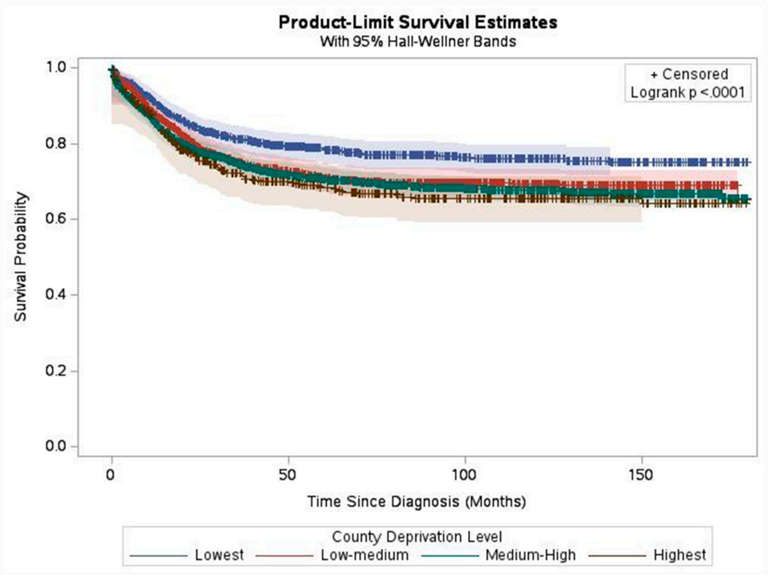

Figure S2b: Patients aged 15-19 years

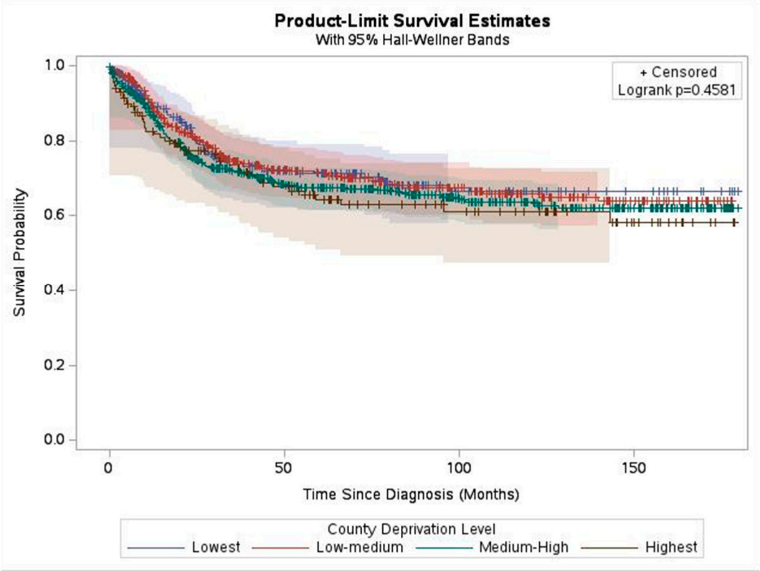

Figure S3. Age-stratified Survival Function by Community Marginalization Status.

Figure S3a: Patients aged 0-14 years

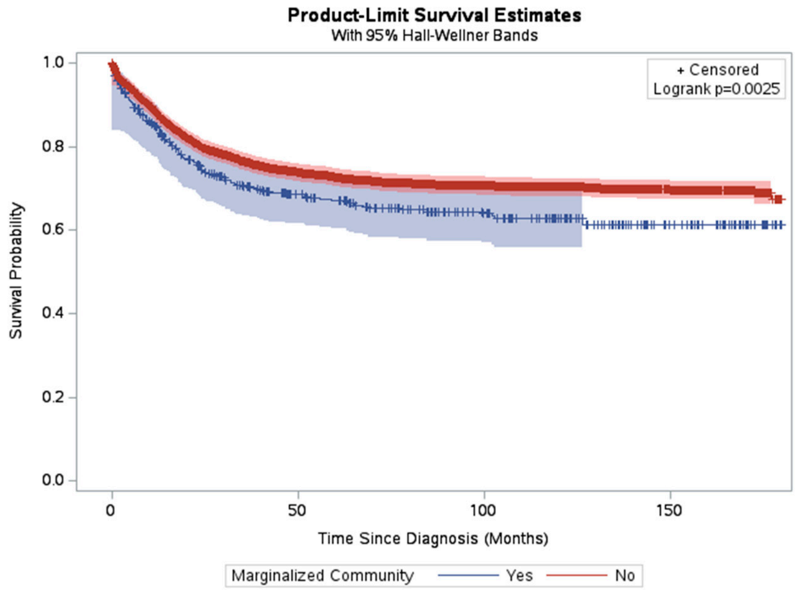

Figure S3b: Patients aged 15-19 years

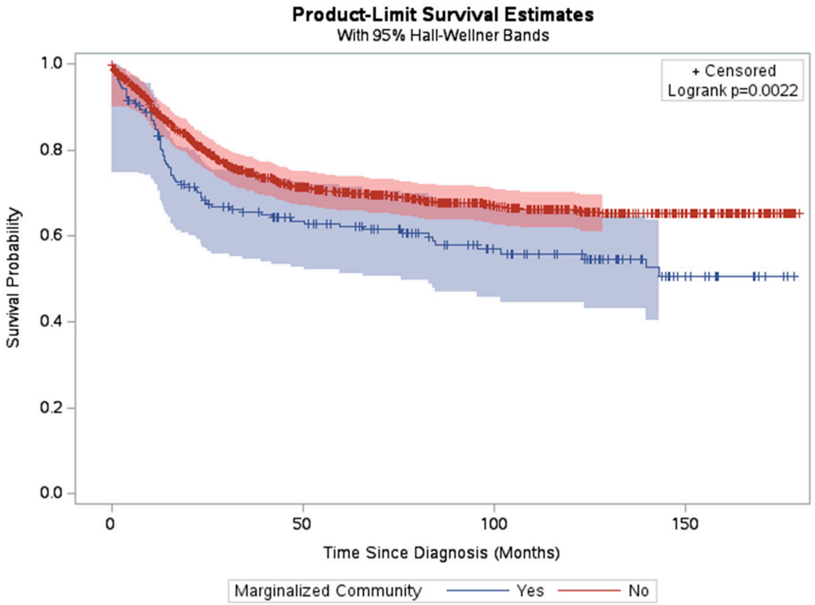

Supplement: Supplementary file 1 [file cancers-18-02627-s001.zip › cancers-4376867-supplementary.pdf]
